# The cortical amygdala consolidates a socially transmitted long-term memory

**DOI:** 10.1038/s41586-024-07632-5

**Published:** 2024-07-03

**Authors:** Zhihui Liu, Wenfei Sun, Yi Han Ng, Hua Dong, Stephen R. Quake, Thomas C. Südhof

**Affiliations:** 1 Department of Molecular and Cellular Physiology, Stanford University School of Medicine, Stanford, CA USA; 2 Howard Hughes Medical Institute, Stanford University School of Medicine, Stanford, CA USA; 3 Department of Bioengineering, Stanford University, Stanford, CA USA; 4 Institute for Stem Cell Biology and Regenerative Medicine, Stanford University School of Medicine, Stanford, CA USA; 5 Chan Zuckerberg Initiative, Redwood City, CA USA

**Keywords:** Neural circuits, Consolidation, Long-term memory

## Abstract

Social communication guides decision-making, which is essential for survival. Social transmission of food preference (STFP) is an ecologically relevant memory paradigm in which an animal learns a desirable food odour from another animal in a social context, creating a long-term memory^1,2^. How food-preference memory is acquired, consolidated and stored is unclear. Here we show that the posteromedial nucleus of the cortical amygdala (COApm) serves as a computational centre in long-term STFP memory consolidation by integrating social and sensory olfactory inputs. Blocking synaptic signalling by the COApm-based circuit selectively abolished STFP memory consolidation without impairing memory acquisition, storage or recall. COApm-mediated STFP memory consolidation depends on synaptic inputs from the accessory olfactory bulb and on synaptic outputs to the anterior olfactory nucleus. STFP memory consolidation requires protein synthesis, suggesting a gene-expression mechanism. Deep single-cell and spatially resolved transcriptomics revealed robust but distinct gene-expression signatures induced by STFP memory formation in the COApm that are consistent with synapse restructuring. Our data thus define a neural circuit for the consolidation of a socially communicated long-term memory, thereby mechanistically distinguishing protein-synthesis-dependent memory consolidation from memory acquisition, storage or retrieval.

## Abstract (web summary)

Experiments in mice show that brain circuitry centred on the posteromedial nucleus of the cortical amygdala has a key role in consolidating socially transmitted long-term memories.

## Main

During social interactions, animals transmit information such as fear, pain and food preferences through sensory and behavioural cues^1–6^. Social transmission of food preference (STFP) serves to convey information about food safety between social conspecifics^1,2^, creating a long-lasting food-odour memory (STFP memory) that overrides innate food preferences. Although STFP memory formation is known to involve multiple brain regions^6–14^, it is unclear how the combination of food odour and social interaction induces STFP memory. The specific roles of various brain regions in different stages of STFP memory formation—memory acquisition, consolidation, storage and recall—are largely unknown, as are the underlying circuits. The accessory olfactory bulb (AOB) and main olfactory bulb (MOB) are likely to mediate social and odour-sensation inputs, respectively, during STFP training, but how their signals are integrated is unclear. The AOB and MOB project to distinct downstream brain regions^15^ that engage in extensive, often reciprocal connections. These connections could integrate olfactory information from the MOB with social information from the AOB, but the precise mechanisms involved have not been studied. Short-term memory is generally thought to be consolidated into long-term memory in at least two phases: an initial molecular consolidation phase that involves a protein-synthesis-dependent mechanism; and a later systems consolidation phase that involves sleep-dependent interactions between the cortex, amygdala and hippocampus^16^. Which circuits and mechanisms mediate memory consolidation, however, and whether such circuits and mechanisms are distinct from those that mediate long-term memory storage and retrieval, remains unclear.

Here we identify a cortical circuit centred on the posteromedial nucleus of the cortical amygdala (COApm) that selectively mediates the early protein-synthesis-dependent phase of STFP memory consolidation without being involved in STFP memory acquisition, storage or retrieval. We show that, in contrast to the ventral hippocampus, which is required for encoding contextual odour-related information^17^, and the orbitofrontal cortex (OFC), which is essential for later phases of STFP memory consolidation and/or retrieval^6^, the COApm circuit is exclusively essential for initial STFP memory consolidation, thus documenting a separable consolidation mechanism for long-term STFP memory. Moreover, we show that STFP memory consolidation involves COApm-specific changes in the expression of genes that encode synaptic proteins, thereby describing the gene-expression architecture of a defined memory consolidation process in an identified circuit.

## STFP training activates COApm neurons

**Fig. 1:**
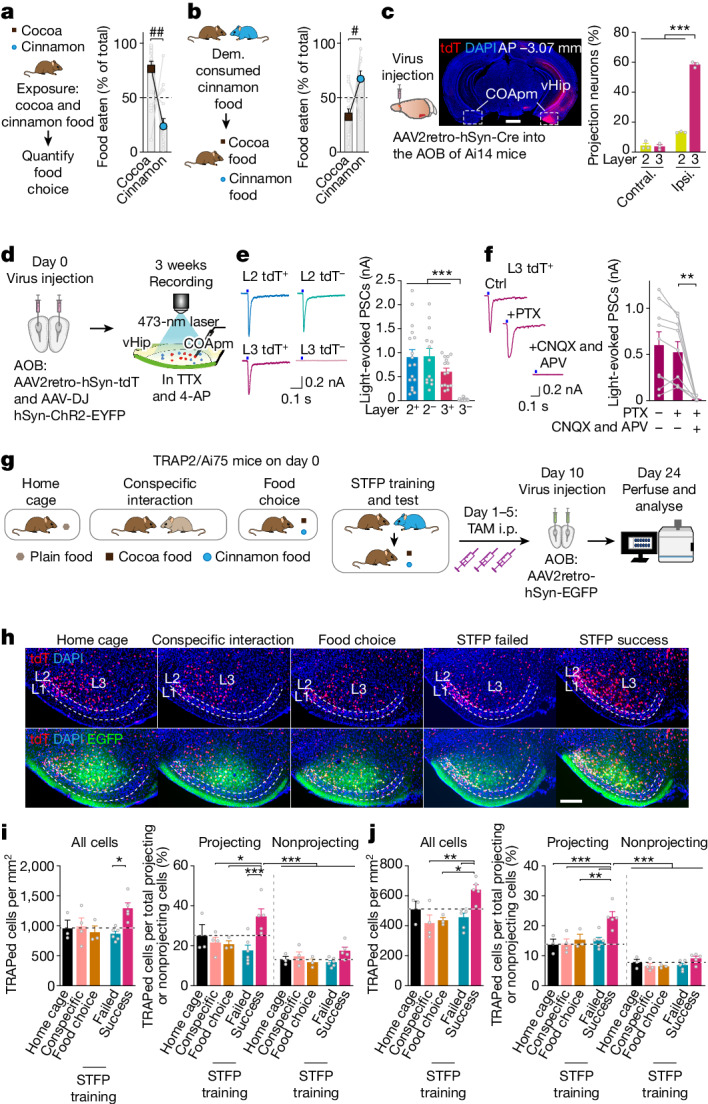
STFP selectively activates neurons in the COApm that form synaptic connections with the AOB. **a**, Innate food preference (*n* = 15 mice, *P* = 0.0043, two-tailed Wilcoxon signed-rank test). **b**, STFP training (*n* = 11 mice, *t*_10_ = 2.464, *P* = 0.0335, two-tailed paired Student’s *t*-test). Dem., demonstrator. **c**, Retrograde tracing showing that COApm neurons project to the AOB (left, schematics; middle, representative image (scale bar, 1 mm); right, percentage of AOB-projecting neurons in the ipsi- and contralateral COApm (*n* = 3 mice; *F*_3,8_ = 523.7, *P* = 1.6 × 10^−9^; one-way ANOVA with post-hoc Tukey test; statistical details are reported in Supplementary Table 6). AP, anterior/posterior to bregma; vHip, ventral hippocampus. **d**–**f**, AOB-projecting COApm neurons receive excitatory inputs from the AOB. **d**, Schematic of experimental strategy. **e**, Sample traces (left) and amplitude (right) of monosynaptic currents (layer 2 (L2): tdT^+^, *n* = 17, tdT^−^, *n* = 13; layer 3 (L3): tdT^+^, *n* = 15, tdT^−^, *n* = 20, cells; *P* = 4.1 × 10^−9^, Kruskal–Wallis with post-hoc two-stage linear step-up test, adjusted *P* value). PSCs, postsynaptic currents. **f**, Optogenetic COApm current inhibition by 6-cyano-7-nitroquinoxaline-2,3-dione (CNQX), D-(-)-2-amino-5-phosphonopentanoic acid (APV) and picrotoxin (PTX) (*n* = 9 cells, for PTX + CNQX + APV versus PTX, *P* = 0.0039, two-tailed Wilcoxon signed-rank test). **g**–**j**, AOB-projecting COApm neurons are selectively activated during long-term STFP memory consolidation. **g**, Schematic of experimental strategy for labelling STFP-training-activated COApm neurons using FOS expression. i.p., intraperitoneal; TAM, tamoxifen. **h**, Representative COApm images (red, TRAPed cells; green, retrogradely labelled COApm–AOB projection neurons). Scale bar, 200 μm. **i**,**j**, Quantification of activated ‘TRAPed’ cell densities in layers 2 (**i**) or 3 (**j**) of all images acquired (left, all neurons; right, AOB-projecting and AOB-nonprojecting neurons) (**g**–**j**: home cage *n* = 3, conspecific *n* = 4, food choice *n* = 3, STFP failed *n* = 6, STFP success *n* = 5 mice; **i** left, *F*_4,16_ = 3.567, *P* = 0.0291; **j** left, *F*_4,16_ = 6.114, *P* = 0.0035, one-way ANOVA with post-hoc Tukey test; **i** right, *F*_4,32_ = 6.337, *P* = 7.1 × 10^−4^; **j** right *F*_4,32_ = 8.749, *P* = 6.9 × 10^−5^; **i**,**j** right, two-way ANOVA with post-hoc Tukey test). All data are mean ± s.e.m. For detailed statistics, see Supplementary Tables 5 and 6; #, *P < 0.05; ##, **P < 0.01; ***P < 0.001. Source Data

**Extended Data Fig. 1:**
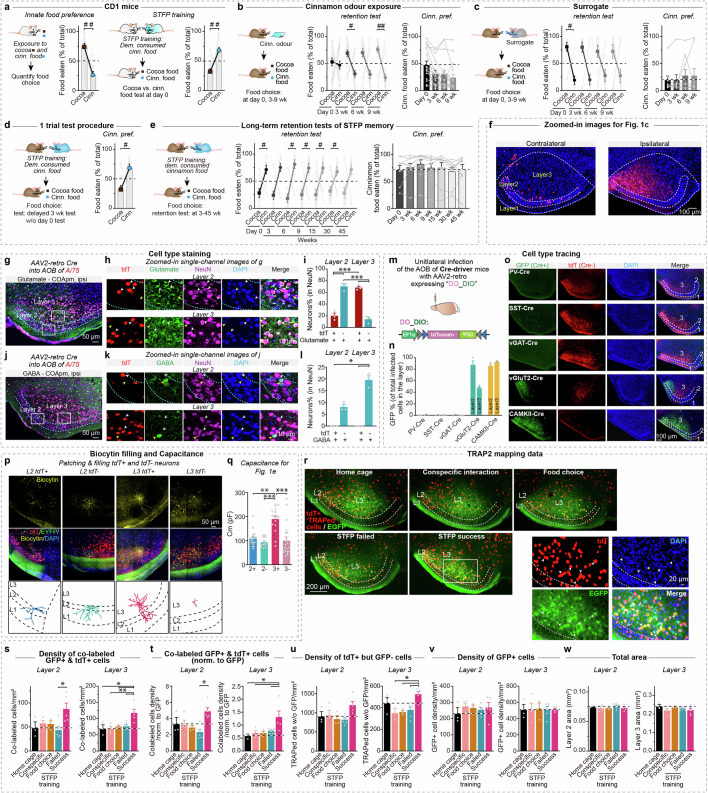
Further characterizations of long-term STFP memory, and description of AOB-projecting neurons in the COApm and TRAP2 mapping of STFP-training-activated neurons in the COApm. **a**, CD1 mice exhibit the same innate food preference as C57BL/6 J mice for cocoa over cinnamon (left) and this innate food preference is similarly reversed by STFP training (right) as shown in Fig. 1a,b for C57BL/6 J mice (for each section, experimental strategies are shown on the left and summary graphs of food consumption on the right [left, innate food-preference measurements, n = 14, t_13_ = 3.825, p = 0.0021; right, STFP measurements, n = 15, t_14_ = 3.680, p = 0.0025, two-tailed paired Student *t*-test]). **b**,**c**. Exposure of C57BL/6 J mice to cinnamon odour alone (**b**) or to a cinnamon-scented fake mouse (**c**) does not alter their innate preference for cocoa over cinnamon different from STFP (left, experimental design; middle and right, percentage of cocoa vs. cinnamon food eaten (middle) or of cinnamon food eaten (right) at different time points [**b**, n = 17, 3 weeks, p = 0.0110; 9 weeks, p = 0.0063; **c**, n = 9, day 0, p = 0.0117, two-tailed Wilcoxon signed-rank test]). **d**, Long-term STFP memory is sustained beyond 3 weeks after a one-trial STFP training session in C57BL/6 J mice even when memory acquisition is not tested on day 0 after STFP training (left, experimental design; right, percentage of food eaten at the 3-week test [n = 10, p = 0.0488, two-tailed Wilcoxon signed-rank test]). **e**, Long-term STFP memory is sustained for at least 45 weeks after a single STFP training session as revealed by retesting the same cohort of mice over a 3-45 week period (left, experimental design; middle and right, percentage of cocoa vs. cinnamon food eaten (middle) or of cinnamon food eaten (right) at different time points [n = 9; day 0, t_8_ = 2.718, p = 0.0263, two-tailed paired Student *t*-test; 6 weeks, p = 0.0117; 9 weeks, p = 0.0273; 15 weeks, p = 0.0273; 30 weeks, p = 0.0391, two-tailed Wilcoxon signed-rank test]). **f**, Expanded representative images of the COApm after unilateral retrograde labelling of COApm neurons using retro-AAVs injected into the AOB (from the boxed areas of Fig. 1c, n = 3 mice). **g**–**l**, Immunohistochemical staining of COApm neurons using antibodies to glutamate (**g**-**i**) or GABA (**j**-**l**) demonstrates that all AOB-projecting COApm neurons are glutamatergic and that only a minority of layer-2 neurons but a majority of layer-3 neurons project to the AOB. AOB-projecting neurons in the COApm were retrogradely labelled by stereotactically infecting the AOB of Ai75 reporter mice with AAV2retro-Cre (**g**,**h**,**j**,**k**, representative images of stained COApm sections [scale bars apply to all images of a set]; **i**,**l**, percentage of glutamate- (**i**) and GABA- positive neurons (**l**) in AOB-projecting tdT+ and AOB-nonprojecting tdT- neurons in layers 2 and 3 [**i**, F_3,8_ = 63.5, p = 6.4 × 10^−6^, one-way ANOVA with Tukey post-hoc test; **l**, p = 0.0006, Kruskal–Wallis with post-hoc Two-stage linear step-up test]; n = 3 mice). **m**–**o**, Cell-type tracing of AOB-projecting neurons of the COApm using defined Cre-driver mouse lines demonstrates that AOB-projecting neurons of the COApm are glutamatergic vGluT2- and CAMKII-expressing neurons, whereas vGAT-, SST- and PV-expressing, presumptively GABAergic, neurons are not labelled (**m**, experimental strategy whereby the AOB of various Cre-driver lines was injected with retro-AAVs encoding tdTomato in the absence and GFP in the presence of Cre to selectively label AOB-projecting neurons with GFP; **n**, quantifications of GFP-positive cells as per cent of total labelled cells [sum of GFP- and tdTomato-positive cells]; **o**, representative images of COApm sections [n = 3 mice for each group]). **p**, Representative images of COApm sections (top) with reconstructed neurons filled with biocytin to map the local dendrites of neurons. Layer 2 tdT+ n = 7, layer 2 tdT- n = 6, layer 3 tdT+ n = 6, layer 3 tdT- n = 3, cells. **q**, Summary graphs of the neuronal capacitance corresponding to the layer-2 and layer-3 tdT+ and tdT- neurons recorded in Fig. 1e (p = 8.03 × 10^−5^, Kruskal–Wallis with post-hoc Two-stage linear step-up test) (layer 2: tdT^+^ , n = 17, tdT-, n = 13; layer 3: tdT^+^, n = 15, tdT-, n = 20, cells). Note that only AOB-projecting tdT+ layer-3 neurons that constitute the vast majority of the AOB-projecting neurons of the COApm exhibit an intrinsically higher capacity, suggesting a larger size. **r**–**v**, Further characterization of TRAP2 mapping of activated COApm neurons in Fig. 1h–j, confirming that only STFP training but not odour by itself or the home cage activates COApm neurons (**r**, merged representative images of TRAPed cells (tdT^+^ , red) and EGFP (green) in COApm sections (top) and expanded single-colour views of sections from STFP-trained mice (bottom), complementing Fig. 1h; **s**–**v**, cell density quantifications of layers 2 and 3 of the COApm, with graphs showing the absolute (**s**) and GFP-normalized (**t**) density of cells co-labelled for GFP+ and tdT+ [**s**, layer 2, F_4,16_ = 3.733, p = 0.0249; layer 3, F_4,16_ = 6.430, p = 0.0028; **t**, layer 2, p = 0.0392 (Kruskal–Wallis with post-hoc Two-stage linear step-up test), layer 3, F_4,16_ = 4.517, p = 0.0124], or showing the density of tdT+ cells lacking GFP (**u**) [layer 3, F_4,16_ = 4.688, p = 0.0107], or showing the total density of GFP+ cells (**v**)). One-way ANOVA with post-hoc Tukey test except for **t** layer 2. **w**, The total area of layers 2 and 3 of the mouse COApm does not change after odour exposure or STFP training. For **s** and **w**, home cage n = 3, conspecific n = 4, food choice n = 3, STFP failed n = 6, STFP success n = 5 mice. Data are means ± s.e.m. For details and statistical comparisons, see Supplementary Tables 5 and 6. *, #p < 0.05, **, ##p < 0.01, ***, ###p < 0.001. Source Data

C57BL/6J or CD1 mice exhibit an innate preference for cocoa- over cinnamon-flavoured food, which is reversed by STFP training^13^ (Fig. 1a,b and Extended Data Fig. 1a). Such reversal could not be induced by exposing mice to cinnamon odour or cinnamon-scented mouse surrogates, suggesting that the social context of STFP training is essential^18^ (Extended Data Fig. 1b,c). STFP memory lasts for months, independent of whether it is tested in a single trial after a prolonged interval or repeatedly in weekly trials^19^ (Extended Data Fig. 1d,e). Thus, STFP is an ecologically relevant one-trial learning paradigm that produces a long-lasting appetitive memory of socially communicated information. Here, we used only male mice, because female mice exhibit oestrous-dependent changes in STFP behaviour^20–22^ (see Supplementary Discussion section 1).

Given its social context, we hypothesized that STFP memory acquisition might not involve only the MOB, which senses odours, but also the AOB, which senses social pheromone signals^23^. The AOB is reciprocally connected to the COApm^24–29^, an enigmatic three-layered cortical nucleus that is implicated in suppressing male mating when a female mouse is unhealthy^4^. Retrograde tracing revealed that most layer-3 neurons of the COApm (around 65%) and a smaller percentage of layer-2 neurons (around 17%) extend ipsilateral excitatory projections to the AOB (Fig. 1c and Extended Data Fig. 1f–o). Optogenetic mapping, in turn, showed that the AOB-projecting layer-3 neurons of the COApm, but not the AOB-nonprojecting neurons, also receive monosynaptic excitatory inputs from the AOB (Fig. 1d–f and Extended Data Fig. 1p,q). In addition, both AOB-projecting and AOB-nonprojecting layer-2 neurons of the COApm receive synaptic AOB inputs. Thus, a feedback circuit connects COApm neurons to the AOB, such that excitation of layer-3 COApm neurons by AOB mitral cells leads to feedback inhibition of these mitral cells through recurrent excitation of AOB granule cells, a notion supported by previous studies^28,30^.

Given the abundant synaptic connections between the AOB and the COApm, we asked whether STFP memory formation activates COApm neurons. We used TRAP2 mice expressing tamoxifen-inducible Cre-ERT2 from the endogenous *Fos* gene, which enables temporally controlled activity-dependent Cre expression^31^. We crossed TRAP2 mice with Ai75 reporter mice that express Cre-dependent tdTomato (tdT) and activated Cre-ERT2 using intraperitoneal tamoxifen injections after STFP training, a time when memories are being consolidated. As controls, we used home cage, scented food only (food choice) or social interactions only (conspecific interaction) conditions combined with tamoxifen injections (Fig. 1g). In all experiments, we retrogradely labelled AOB-projecting COApm neurons using AOB infections with EGFP-expressing retro-AAVs to determine whether activated tdT^+^ neurons project back to the AOB (Fig. 1g).

Successful STFP training, but not STFP training failures, strongly activated AOB-projecting but not AOB-nonprojecting COApm neurons in layers 2 and 3 (Fig. 1h–j and Extended Data Fig. 1r–w). Neither scented food alone nor social interactions alone activated COApm neurons above home-cage backgrounds. These results suggest that STFP training, but not olfaction or social interaction alone, stimulates AOB–COApm projections.

## STFP memory formation requires the COApm

**Fig. 2:**
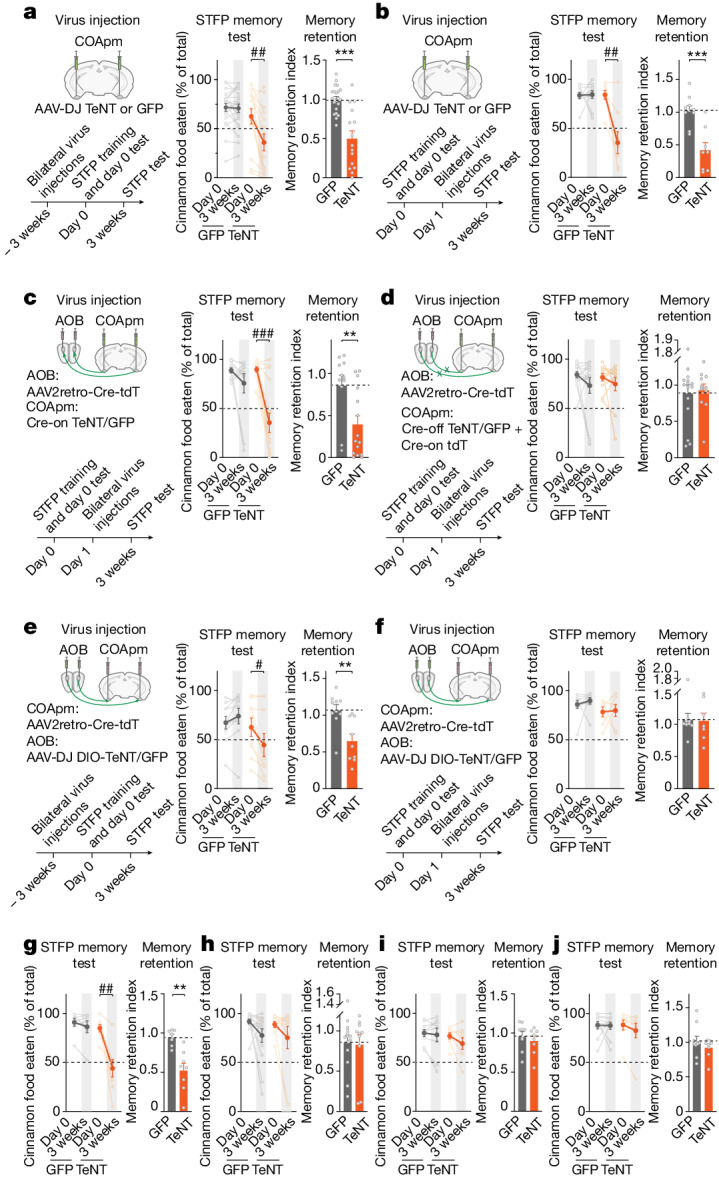
Silencing of COApm or OFC neurons, but not of BLA, ventral hippocampus or mPFC neurons, blocks long-term STFP memory formation. All panels analyse the effects of the indicated manipulations on STFP memory formation, with **a**–**f** depicting the experimental strategy on the left and summary graphs on the right and **g**–**j** following the same strategy as **b**. **a**,**b**, TeNT silencing of the COApm three weeks before (**a**) or one day after (**b**) training (**a**, GFP, *n* = 22, TeNT, *n* = 15; middle, *P* = 0.0012; right, *P* = 5.4 × 10^−5^; **b**, GFP, *n* = 10, TeNT, *n* = 8; middle, *t*_7_ = 4.374, *P* = 0.0033; right, *t*_16_ = 4.626, *P* = 2.8 × 10^−4^). **c**,**d**, TeNT silencing of AOB-projecting (**c**), but not of AOB-nonprojecting (**d**) COApm neurons impairs long-term STFP memory (**c**, GFP, *n* = 11, TeNT, n = 15, middle, *P* = 4.3 × 10^−4^, right, *P* = 0.0090; **d**, GFP, *n* = 15, TeNT, *n* = 11). **e**,**f**, TeNT silencing of AOB neurons projecting to the COApm instituted three weeks before (**e**) or 1 day after (**f**) STFP training (**e**, GFP, *n* = 10, TeNT, *n* = 9; **e** middle, *P* = 0.0195; **e** right, *t*_17_ = 3.447, *P* = 0.0031; **f**, GFP, *n* = 9; TeNT, *n* = 7). **g**–**j**, TeNT silencing one day after STFP training in the OFC (**g**), ventral hippocampus (**h**), BLA (**i**) or mPFC (**j**) (**g**, GFP *n* = 7, TeNT *n* = 8, left, *t*_7_ = 4.774, *P* = 0.0020, right, *P* = 0.0012; **h**, GFP *n* = 14, TeNT *n* = 10; **i**, GFP *n* = 9, TeNT *n* = 8; **j**, GFP *n* = 10, TeNT *n* = 8). Data are mean ± s.e.m. Statistics: two-tailed paired Student’s *t*-test: **b**,**f**,**g**,**i** (middle-TeNT); two-tailed unpaired Student’s *t*-test: **b**,**e**,**i** (right); two-tailed Wilcoxon signed-rank test: **a**,**c**,**d**,**e**,**h**,**j** (middle), **b**,**f**,**i** (middle-GFP); two-sided Mann–Whitney test: **a**,**c**,**d**,**f**,**g**,**h**,**j** (right), with #, *P < 0.05; ##, **P < 0.01; ###, ***P < 0.001. For detailed statistics, see Supplementary Tables 5 and 6. Source Data

**Extended Data Fig. 2:**
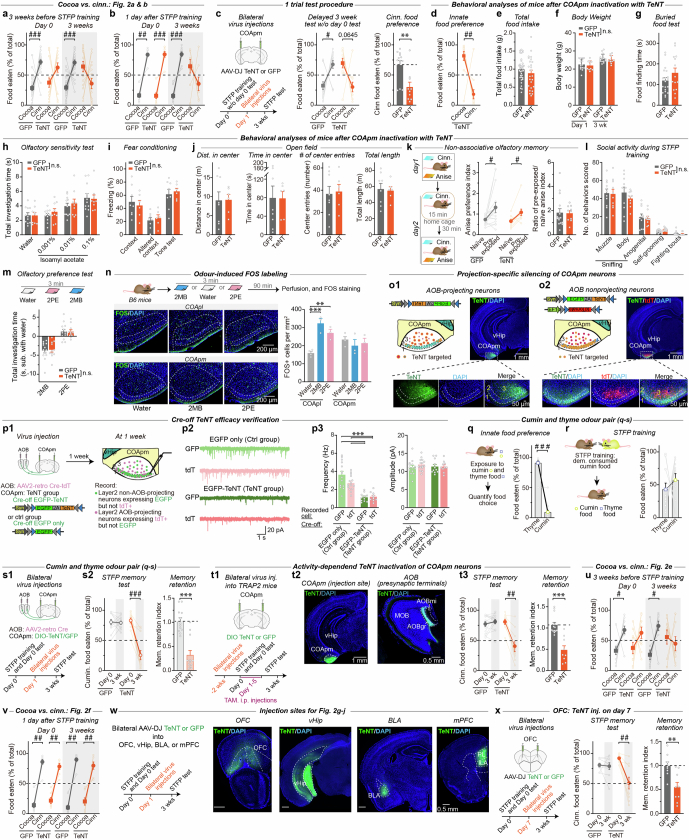
Experiments including extensive further controls to show that TeNT-induced silencing of COApm neurons specifically and efficiently impairs long-term STFP memory. **a**,**b**, Percentage of food eaten at day 0 and 3 weeks after STFP training (**a**, AAVs-TeNT-EGFP or EGFP were injected 3 weeks prior to STFP training in Fig. 2a [Day 0 GFP, p = 0.0003; 3 weeks, GFP, t_21_ = 4.854, p = 8.5 × 10^−5^, GFP, n = 22, TeNT, n = 15]; **b**, mice with successful STFP training were injected with the same AAVs one day after STFP training in Fig. 2b [day 0, GFP, p = 0.0020, TeNT, t_7_ = 7.309, p = 0.0002; 3 weeks, GFP, t_9_ = 8.721, p = 1.1 × 10^−5^], GFP, n = 10, TeNT, n = 8). **c**, TeNT silencing of the COApm after STFP training also blocks long-term STFP memory when using a one-trial test procedure that omits tests of short-term STFP memory acquisition on day 0 (left, experimental strategy; middle, percentage of cocoa- and cinnamon-flavoured food consumed at 3 weeks; right, percentage of cinnamon-flavoured food eaten at 3 weeks in the middle [GFP, n = 14; TeNT, n = 10; middle, cocoa vs. cinnamon, GFP, p = 0.0295; TeNT, n = 0.0645; right, p = 0.0073]). **d**–**j**, TeNT-induced silencing of the COApm has no effect on innate food preference (**d**), total food intake (**e**), body weight (**f**), buried food finding test (**g**), olfactory sensitivity (**h**), contextual fear memory (**i**) and open field behaviours of mice (**j**), demonstrating its selectivity for long-term STFP memory formation without altering olfaction (**d**, n = 12, p = 0.0068; **e**, GFP, n = 36; TeNT, n = 25; **f**, GFP, n = 14; TeNT, n = 10; **g**, GFP, n = 19; TeNT, n = 14; **h**, GFP, n = 11; TeNT, n = 9; **i**, GFP, n = 8; TeNT, n = 6; **j**, GFP, n = 8; TeNT, n = 6). **k**, TeNT silencing of the COApm does not impair non-associative olfactory memory (left, experimental strategy; middle, anise preference index of naive versus pre-exposed mice does not differ between GFP (p = 0.0181) vs. TeNT groups (p = 0.0137); right, ratio of pre-exposed anise preference index/naive anise preference index [GFP, n = 15; TeNT, n = 10]). **l**, Quantifications reveal that TeNT silencing of COApm neurons does not alter social behaviours (observers’ sniffing at demonstrators’ muzzle, body, and anogenital areas, observers’ self-grooming, and total fighting bouts between observers and demonstrators as scored during the 30-minute social interaction phase of STFP training [GFP, n = 11; TeNT, n = 10]). **m**, TeNT-mediated silencing of COApm neurons has no effect on odour preferences using aversive and attractive odour pairs. Aversive odour, 2MB, 2-methylbutyric acid; and attractive odour, 2PE, 2-phenylethanol (GFP, n = 13; TeNT, n = 11). **n**, COApm neurons are not directly activated by aversive or attractive odours as analysed by FOS immunohistochemistry, whereas COApl neurons fully respond, thus constituting a positive control^35^ (top; experimental strategy; bottom left, sample images of the COApl; bottom right, summary graph of FOS+ neurons [COApl, F_2,7_ = 22.10, p = 9.4 × 10^−4^; water n = 4, 2MB n = 3, 2PE n = 3, mice]). **o**, Representative injection site images (right) and schematic of AAV constructs (left) for Fig. 2c,d (**o1**, Cre-on EGFP & TeNT; **o2**, Cre-off EGFP & TeNT combined with Cre-on tdT). **p**, mEPSC recordings in layer 2 of the COApm demonstrate that Cre-off TeNT expression effectively silences non-AOB-projecting neurons. Recordings were performed in AOB-projecting or non-AOB-projecting neurons, both of which receive local synaptic inputs from non-AOB-projecting neurons expressing control proteins or TeNT (**p1**, experimental strategy; **p2**, example traces; **p3**, mEPSC frequency (left) and amplitude (right) summary graphs in the four conditions of **p1** and **p2** [left, p = 6.4 × 10^−7^; EGFP only set: GFP+ n = 14/4, tdT^+^ , n = 15/4; EGFP-TeNT set: GFP+ , n = 11/3, tdT^+^ , n = 10/4, cells/mice]). **q**–**s**, Repeat of the experiments in Figs. 1a,b and 2b with a different food odour pair (cumin vs. thyme) demonstrates that C57BL/6 J mice exhibit an innate food preference for thyme (**q**) that can be reversed by STFP training^38^ (**r**), and that with this food odour pair TeNT-induced silencing of COApm AOB-projecting neurons after STFP training also inactivates long-term STFP memory formation (**s**). Mice used in **s** included successfully trained mice in **r** (**q** & **r**: left, experimental strategy; right, summary graph of percentage of food eaten [**q**, n = 15, p = 1.2 × 10^−4^; **r**, n = 11]; **s1**, injection strategy; **s2** left, percentage of cumin-flavoured food eaten; **s2** right, memory retention index [GFP, n = 12, TeNT, n = 12, with TeNT in the left graph, p = 9.8 × 10^−4^; right graph, p = 1.4 ×10^−4^]). **t**, Selective activity-dependent TeNT-induced silencing of COApm neurons using TRAP2 mice severely impairs long-term STFP memory (**t1**, experimental strategy; **t2**, injection sites of COApm (top) and their projections to the AOB (bottom); **t3**, percentage of cinnamon-flavoured food on day 0 and 3 weeks (left) and memory retention index (right) [GFP, n = 14; TeNT, n = 9. **t3**, left, TeNT, t_8_ = 5.004, p = 0.0010; right, p = 1.1 × 10^−4^]). **u**,**v**, Percentage of food eaten at day 0 and 3 weeks after STFP training (see Fig. 2e,f) (**u**, GFP, n = 10, TeNT, n = 9; day 0, GFP, t_9_ = 2.662, p = 0.0260; 3 weeks, GFP, p = 0.0273. **v**, GFP, n = 9; TeNT, n = 7; day 0, GFP, p = 0.0039; TeNT, t_6_ = 5.658, p = 0.0013; 3 weeks, GFP, p = 0.0039; TeNT, t_6_ = 4.973, p = 0.0025). **w**, Experimental strategy (left) and example images of injection sites (right) for experiments in Fig. 2g–j. **x**, TeNT silencing of OFC neurons 7 days after STFP training impairs long-term STFP memory (left, experimental strategy; middle, percentage of cinnamon-flavoured food on day 0 and after 3 weeks; right, memory retention index [GFP, n = 9; TeNT, n = 9. Middle, TeNT, t_8_ = 4.495, p = 0.0020; right, t_16_ = 3.527, p = 0.0028]). Data are means ± s.e.m. Statistics: two-tailed paired student *t*-test: **a** (3 weeks-GFP), **b** (day 0-TeNT, 3 weeks-GFP), **t3** (left-TeNT), **u** (day 0-GFP), **v** (day 0-TeNT, 3 weeks-TeNT), **x** (middle-TeNT); two-tailed unpaired student *t*-test: **x** (right); two-tailed Wilcoxon signed-rank test: **a** (day 0-GFP), **b** (day 0-GFP), **c** (middle), **d**, **k**, **q**, **s2** (left-TeNT), **u** (3 weeks), **v** (day 0-GFP, 3 weeks-GFP); two-tailed Mann–Whitney test: **c** (right), **t3** (right), **s2** (right); one-way ANOVA with Tukey post-hoc test: **n** (COApl); Kruskal–Wallis with post-hoc Two-stage linear step-up test: **p3** (left-frequency). For details and statistical comparisons, see Supplementary Tables 5 and 6. #, *p < 0.05, ##, **p < 0.01, ###, ***p < 0.001. Source Data

Next, we asked whether COApm activity is required for STFP memory formation. We silenced all synaptic signalling of COApm neurons using AAVs expressing tetanus toxin light chain (TeNT), which blocks neurotransmitter release by cleaving synaptobrevins^32–34^. TeNT-induced silencing of the COApm before STFP training completely abolished long-term STFP memory measured three weeks after training, but had no significant effect on recent STFP memories measured on the day of training (Fig. 2a and Extended Data Fig. 2a). Notably, TeNT-induced silencing of the COApm one day after STFP training also abolished long-term STFP memory measured three weeks after training, independent of whether or not recent STFP memories were tested on the day of STFP training (Fig. 2b and Extended Data Fig. 2b,c). TeNT-induced silencing of the COApm did not alter body weights, food consumption or behavioural parameters such as social interactions, open field activity or fear conditioning. Silencing the COApm also did not impair olfaction as monitored by odorant sensitivity, odour preference, innate food preference, buried food tests or a non-associative olfactory memory task (Extended Data Fig. 2d–m and Supplementary Table 1). Moreover, measurements of olfactory responses using FOS staining showed that the COApm was not activated by aversive or attractive odours alone, in contrast to the adjacent posterolateral cortical amygdala (COApl), which is known to sense odours^35^ (Extended Data Fig. 2n). Together, these results reveal that the COApm is not required for olfaction, social interactions, olfactory learning or STFP memory acquisition, but is selectively essential for long-term STFP memory formation.

Because a subset of COApm neurons are connected to, and activated by, AOB neurons (Fig. 1), we asked whether AOB inputs into the COApm govern STFP memory formation. We first investigated the role of AOB-projecting versus AOB-nonprojecting COApm neurons in long-term STFP memory formation using selective silencing of these COApm neuron subsets. We injected retro-AAVs expressing Cre into the AOB and AAVs expressing Cre-inducible (‘Cre-on’) or Cre-blockable (‘Cre-off’) TeNT into the COApm, thereby selectively inactivating AOB-projecting or AOB-nonprojecting neurons, respectively. Long-term STFP memory tests showed that only AOB-projecting but not AOB-nonprojecting COApm neurons were required for long-term STFP memory formation (Fig. 2c,d and Extended Data Fig. 2o). Electrophysiological measurements validated the effectiveness of the Cre-off TeNT-induced silencing of AOB-nonprojecting neurons (Extended Data Fig. 2p). Furthermore, we confirmed with a different odour pair—cumin versus thyme—that AOB-projecting COApm neurons are required for long-term STFP memory formation, demonstrating that the COApm acts in long-term STFP memory formation independent of odour (Extended Data Fig. 2q–s). Moreover, selective inactivation of COApm neurons that are activated during long-term STFP memory formation by stereotactically injecting TRAP2 mice with AAVs encoding Cre-dependent TeNT and inducing Cre-ERT2 after successful STFP training using tamoxifen also ablated long-term STFP memory (Extended Data Fig. 2t). Thus, only COApm neurons that are synaptically connected with the AOB are required for long-term STFP memory formation.

We next investigated whether the AOB input into the COApm is required for long-term STFP memory formation. We addressed this question by injecting the COApm with retro-AAVs expressing Cre and the AOB with AAVs expressing Cre-on TeNT. Silencing COApm-projecting AOB neurons with this approach impaired long-term STFP memory formation when instituted before STFP training, but not when performed after training (Fig. 2e,f and Extended Data Fig. 2u,v)—different from COApm neurons, the silencing of which after STFP training still blocked long-term STFP memory formation (Fig. 2b). Thus, the AOB–COApm projection is essential for long-term STFP memory formation only during memory acquisition, whereas the COApm itself is required after memory acquisition.

Several brain regions have been implicated in STFP memory formation, including the OFC^6,10,11^, medial prefrontal cortex (mPFC)^10,11,36–38^, ventral hippocampus^6,10–12,37,39–41^ and basolateral amygdala (BLA)^42,43^. TeNT silencing of these brain regions one day after STFP training revealed that, besides the COApm, only the OFC is required for long-term STFP memory, whereas the ventral hippocampus, BLA and mPFC are not (Fig. 2g–j and Extended Data Fig. 2w). Consistent with previous studies^6,10,11^, silencing the OFC seven days after STFP training also impaired long-term STFP memory (Extended Data Fig. 2x).

## The COApm consolidates STFP memory

**Fig. 3:**
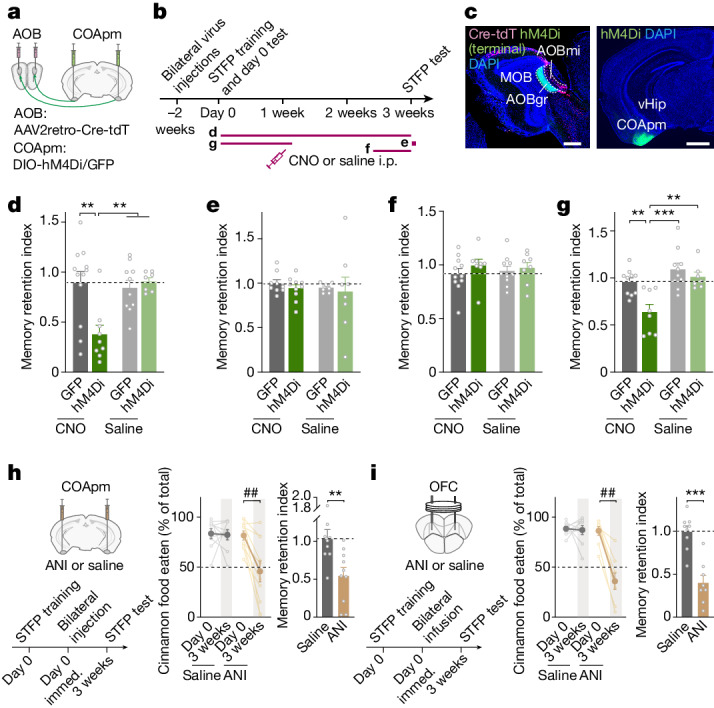
AOB-projecting COApm neurons mediate long-term STFP memory consolidation through protein synthesis. **a**–**c**, Experimental chemogenetics approach for silencing of COApm AOB-projecting neurons. **a**, Injection strategy. **b**, Timeline (letters refer to panels **d**–**g**). **c**, Representative sagittal AOB (left) and coronal (right) brain sections (red, Cre-tdTomato expressed via retro-AAVs injected into the AOB; green, DIO-hM4Di-GFP in the COApm and transported to AOB axon terminals). AOBgr, AOB granule cells; AOBmi, AOB mitral cells. Scale bars, 0.5 mm (left); 1 mm (right). **d**–**g**, Effect of temporally controlled chemogenetic suppression of COApm neuron activity. CNO was administered for the entire three weeks (**d**), 40 min before test (**e**), during the third week (**f**) or during the first week (**g**) (**d**, CNO-GFP, *n* = 12 mice; CNO-hM4Di, *n* = 9; saline-GFP, *n* = 10; saline-hM4Di, *n* = 7; *F*_3,34_ = 6.985, *P* = 8.7 × 10^−4^; **e**, CNO-GFP, *n* = 9; CNO-hM4Di, *n* = 10; saline-GFP, *n* = 7; saline-hM4Di, *n* = 8; **f**, CNO-GFP, *n* = 11; CNO-hM4Di, *n* = 8; saline-GFP, *n* = 10; saline-hM4Di, *n* = 8; **g**, CNO-GFP, *n* = 10; CNO-hM4Di, *n* = 8; saline-GFP, *n* = 10; saline-hM4Di, *n* = 7; *F*_3,31_ = 9.772, *P* = 1.1 × 10^−4^). **h**,**i**, Effect of anisomycin (ANI) administered into the COApm (**h**) or OFC (**i**) immediately (immed.) after STFP training (day 0). Left, experimental design; middle, percentage of cinnamon-flavoured food eaten on day 0 and after 3 weeks; right, memory retention indices (**h**, saline *n* = 9, ANI *n* = 10, middle, *t*_9_ = 3.888, *P* = 0.0037; right, *t*_17_ = 3.002, *P* = 0.0080; **i**, saline *n* = 9, ANI *n* = 8, middle, *P* = 0.0078, right, *t*_15_ = 5.486, *P* = 6.3 × 10^−5^). Data are mean ± s.e.m. Statistics: two-tailed paired Student’s *t*-test: **h** (middle), **i** (middle-saline); two-tailed unpaired Student’s *t*-test: **h**,**i** (right); two-tailed Wilcoxon signed-rank test: **i** (middle-ANI); one-way ANOVA with Tukey post-hoc test: **d**,**g**; Kruskal–Wallis with post-hoc two-stage linear step-up test: **e**,**f**. #, *P < 0.05; ##, **P < 0.01; ###, ***P < 0.001. For detailed statistics, see Supplementary Tables 5 and 6. Source Data

**Extended Data Fig. 3:**
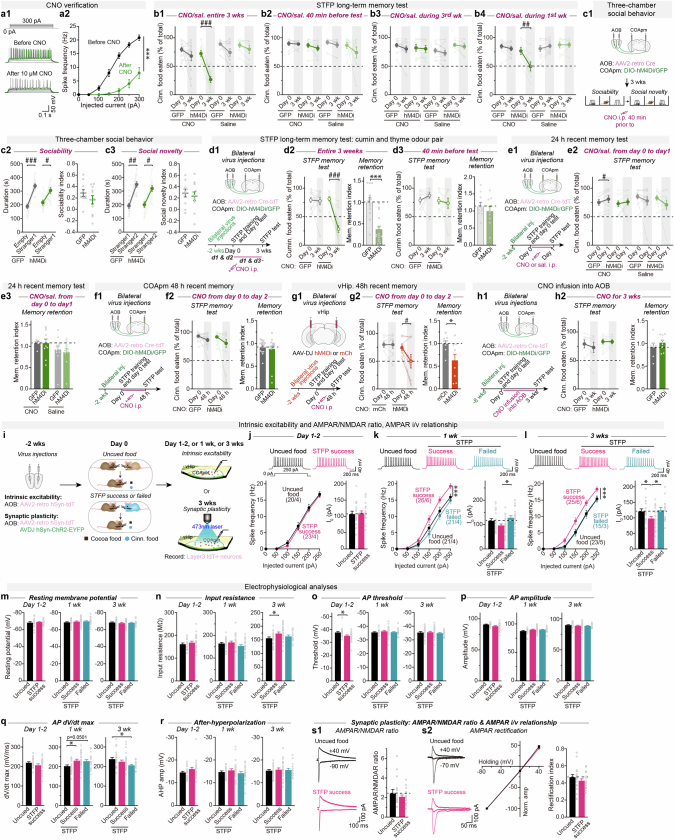
Further experimental data for temporally defined COApm-silencing experiments using chemogenetics and electrophysiological analyses of long-term STFP memory. **a**, Validation of the efficacy of chemogenetic silencing of COApm neurons in successfully STFP-trained mice (**a1**, example traces [green trace = 200 pA current injection]; **a2**, spike frequency before and after perfusion of 10 μM CNO [n = 12 cells, F_1,154_ = 196.2, p < 1.0 × 10^−15^]). **b**, Summary graphs of the cinnamon-flavoured food consumption in STFP memory tests on day 0 and at 3 weeks during the chemogenetics experiments (corresponds to Fig. 3d–g, **b1**, CNO-GFP, n = 12 mice; CNO-hM4Di, n = 9; saline-GFP, n = 10, saline-hM4Di, n = 7; **b2**, CNO-GFP, n = 9; CNO-hM4Di, n = 10; saline-GFP, n = 7, saline-hM4Di, n = 8; **b3**, CNO-GFP, n = 11; CNO-hM4Di, n = 8; saline-GFP, n = 10, saline-hM4Di, n = 8; **b4**, CNO-GFP, n = 10; CNO-hM4Di, n = 8; saline-GFP, n = 10, saline-hM4Di, n = 7; [**b1**, CNO, hM4Di, 3 wk vs. day 0, t_8_ = 6.927, p = 1.2 × 10^−4^; **b4**, CNO, hM4Di, 3 wk vs. day 0, t_7_ = 4.092, p = 0.0046]). **c**, Chemogenetic silencing of the COApm has no effect on three-chamber social behaviours (**c1**, experimental strategy; **c2** & **c3**, summary plots of sociability (**c2**) and social novelty behaviours (**c3**), with left and right graphs showing the durations and mean ratios (indices) of interactions [GFP, n = 10; hM4Di, n = 11; **c2**, left, GFP, t_9_ = 4.840, p = 9.2 × 10^−4^; hM4Di, t_10_ = 2.280, p = 0.0458; **c3**, left, GFP, t_9_ = 4.083, p = 0.0027; hM4Di, t_10_ = 2.953, p = 0.0145]). **d**, Chemogenetic silencing of AOB-projecting COApm neurons, when applied during the 3 weeks after STFP training but not when applied during the 40 min before the 3-week long-term STFP memory test, also blocks STFP memory formation that is tested with the cumin vs. thyme food odour pair different from the cinnamon vs. cocoa food odour pair used in analogous experiments in Fig. 3 (**d1**, experiment strategy; **d2** & **d3** left, percentage of cumin food consumed on day 0 and after 3 weeks; **d2** & **d3** right, memory retention indices [**d2**, GFP, n = 13; hM4Di, n = 16, left, hM4Di, p = 3.1 × 10^−5^; right, p = 6.0 × 10^−5^; **d3**, GFP, n = 10; Gi, n = 9]). **e**,**f**, Chemogenetic silencing of AOB-projecting COApm neurons for 24 h (**e**) or for 48 h (**f**) after STFP training does not impair recent STFP memory formation (**e1** & **f1**, experimental strategies; **e2** & **f2** left, percentage of cinnamon food consumed on day 0 and day 1 or 2; **e2** & **f2** right, memory retention indices [**e2**, CNO-GFP, n = 8; CNO-hM4Di, n = 11; saline-GFP, n = 12; saline-hM4Di, n = 10, CNO, GFP, day 1 vs. day 0, t_7_ = 2.474, p = 0.0426; **f2**, GFP, n = 16; Gi, n = 15]). A saline control was only performed for the 24 h but not the 48 h test since the 24 h chemogenetic inhibition had no effect on recent STFP memory formation. **g**, Chemogenetic silencing of the ventral hippocampus for 48 h after STFP training significantly impairs recent STFP memory formation (**g1**, experimental strategy; **g2** left, percentage of cinnamon-flavoured food eaten on day 0 and day 2; **g2** right, memory retention index [mCh, n = 9; Gi, n = 9; g2 left, hM4Di, t_8_ = 2.782, p = 0.0239; g2 right, p = 0.0315]). **h**, Chemogenetic silencing of COApm-derived presynaptic terminals in the AOB, implemented by CNO infusions for 3 weeks after STFP training, doesn’t alter long-term STFP memory formation, thereby confirming TeNT-silencing experiments showing that the AOB is only involved in STFP memory acquisition but not consolidation (**h1**, experimental strategy; **h2**, left, percentage of cinnamon-flavoured food eaten; right, memory retention index [GFP, n = 7; hM4Di, n = 9]). **i**, Experimental strategy for electrophysiological analyses. **j**–**l**, Successful STFP training does not alter the intrinsic excitability of AOB-projecting COApm neurons at 1–2 days after STFP training (**j**) but produces a significant shift at 1 week (**k**) or 3 weeks after STFP training (**l**) (**j**–**l** top, example traces; **j**–**l** bottom left, summary plots of the spike frequency as a function of injected current; **j**–**l** bottom right, summary graphs of the calculated current required to elicit minimal spiking (I_0_) [day 1–2, uncued food, n = 20/4, STFP success, n = 23/4; 1 week, uncued food, n = 21/4, STFP success, n = 26/6, STFP failed, n = 21/4; 3 week, uncued food, n = 23/5, STFP success, n = 25/6, STFP failed, n = 15/3, cells/mice; **k** lower left, F_2, 390_ = 20.06, p = 5.1 × 10^−9^, STFP success vs. uncued food p = 8.9 × 10^−5^, STFP success vs. STFP failed p = 7.2 × 10^−9^; **k** lower right, F_2, 65_ = 4.426, p = 0.0158; **l** lower left, F_2, 360_ = 16.84, p = 1.0 × 10^−7^, STFP success vs. uncued food p = 2.8 × 10^−6^, STFP success vs. STFP failed p = 7.1 × 10^−6^; **l** lower right, F_2, 60_ = 4.212, p = 0.0194]). **m**–**r**, Successful STFP training does not alter the resting membrane potential (**m**), input resistance (**n**), firing threshold (**o**), amplitude (**p**), or after-hyperpolarization amplitude (**r**) of layer-3 AOB-projecting COApm neurons at 1–2 days, 1 week or 3 weeks after STFP training and does not affect the axon potential rise time (AP dV/dt max) at 1–2 days after STFP training (**q** left) but modestly increases this parameter at 1 week after STFP training (**q** middle, F_2, 65_ = 4.316, p = 0.0174) and decreases it at 3 weeks after STFP training (**q** right, F_2, 60_ = 3.040, p = 0.0553) (n’s are the same as in **j**–**l**, day 1–2, uncued food, n = 20/4, STFP success, n = 23/4; 1 week, uncued food, n = 21/4, STFP success, n = 26/6, STFP failed, n = 21/4; 3 week, uncued food, n = 23/5, STFP success, n = 25/6, STFP failed, n = 15/3, cells/mice; [**n**, right, F_2,60_ = 3.114, p = 0.0517; **o**, left, p = 0.0228]). **s**, Successful STFP training does not alter the AMPAR/NMDAR ratio (**s1**) or the rectification index of AMPAR EPSCs (**s2**) of layer-3 AOB-projecting COApm neurons (**s1** left, example traces; **s1** right, AMPAR/NMDAR ratio summary graph; **s2** left, example traces; **s2** right, AMPAR rectification index summary graph [**s1**, uncued n = 17/4, STFP success n = 17/4; **s2**, uncued n = 19/4, STFP success n = 17/4, cells/mice]). Data are means ± s.e.m. Statistics: two-tailed paired student *t*-test: **b1** (CNO-hM4Di), **b4** (CNO-hM4Di), **c2** (left), **c3** (left), **e2** (CNO-GFP), **g2** (left-hM4Di); two-tailed Wilcoxon signed-rank test: **d**2 (left); two-sided Mann–Whitney test: **d**2 (right), **g**2 (right), **o** (left); one-way ANOVA with Tukey post-hoc test: **k** and **l** (lower right), **q** (middle and right), **n** (right); two-way ANOVA: **a2**, **k** and **l** (lower left). For details, see Supplementary Tables 5 and 6. #, *p < 0.05, ##, **p < 0.01, ###, ***p < 0.001. Source Data

We next sought to understand whether the COApm’s essential role in long-term STFP memory formation operates in memory consolidation, storage or retrieval. To address this question, we inhibited AOB-projecting COApm neurons in a temporally controlled manner using chemogenetics with hM4Di, an inhibitory receptor activated by clozapine *N*-oxide (CNO)^44^ (Fig. 3a–c and Extended Data Fig. 3a). Chemogenetic suppression of COApm neurons for three weeks after STFP training blocked long-term STFP memory (Fig. 3d and Extended Data Fig. 3b). However, chemogenetic suppression of COApm neurons applied during the STFP memory test at the end of week 3 or during the last week before the three-week STFP memory test did not impair long-term STFP memory (Fig. 3e,f and Extended Data Fig. 3b). By contrast, suppressing COApm neuron activity during the first week after STFP training also potently blocked long-term STFP memory (Fig. 3g and Extended Data Fig. 3b). Chemogenetic suppression of COApm neurons did not impair social behaviours (Extended Data Fig. 3c). CNO administration to mice expressing only GFP or saline administration to mice expressing hM4Di had no effect on STFP memory (Fig. 3 and Extended Data Fig. 3b). Moreover, chemogenetic suppression of the COApm after STFP training with the cumin versus thyme food pair also blocked long-term STFP memory, confirming a general role of the COApm in memory consolidation independent of the odour pair (Extended Data Fig. 3d). Thus, activity of COApm neurons is selectively required for long-term STFP memory formation during the first week after STFP training, which suggests that the COApm has a role only in memory consolidation and not in memory storage or retrieval.

The robust impairment of long-term STFP memory by a one-week suppression of COApm neuron activity after STFP training (Fig. 3g) raises the question of whether the COApm might, after all, be involved in STFP memory acquisition, which we might have missed when we routinely tested STFP memory acquisition immediately after STFP training. We therefore examined the effect of a 24-h or 48-h chemogenetic suppression of COApm neurons on STFP memory acquisition, but observed no effect on STFP memories (Extended Data Fig. 3e,f). By contrast, chemogenetic silencing of the ventral hippocampus for 48 h after STFP training significantly impaired STFP memory acquisition (Extended Data Fig. 3g). Thus, the COApm is indeed dispensable for STFP memory acquisition, whereas the ventral hippocampus is essential, consistent with FOS expression data^10–12^. Furthermore, suppression of the activity of COApm terminals in the AOB after STFP training did not impair long-term STFP memory (Extended Data Fig. 3h). Consistent with the TeNT-silencing experiments of the AOB (Fig. 2f and Extended Data Fig. 2v), AOB–COApm connections do not contribute to long-term memory formation after memory acquisition.

Finally, we asked whether memory consolidation by the COApm affects its electrophysiological properties. We injected the AOB of mice with retro-AAVs expressing tdTomato before STFP training or control treatments (Extended Data Fig. 3i). Subsequent current-clamp recordings from layer-3 AOB-projecting COApm neurons in acute slices showed that neurons from mice with successful STFP training, but not from mice with unsuccessful STFP training or uncued controls, exhibited a significant increase in intrinsic excitability at one and three weeks after STFP training without changes in passive electrical properties or action potential parameters (Extended Data Fig. 3j–r). Parallel optogenetic measurements of synaptic responses mediated by AOB–COApm projections did not reveal changes in the AMPA/NMDA ratio or in the *I*/*V* relationship of AMPA-mediated excitatory postsynaptic currents (EPSCs) (Extended Data Fig. 3s).

## Spatial transcriptomics of STFP memory

**Fig. 4:**
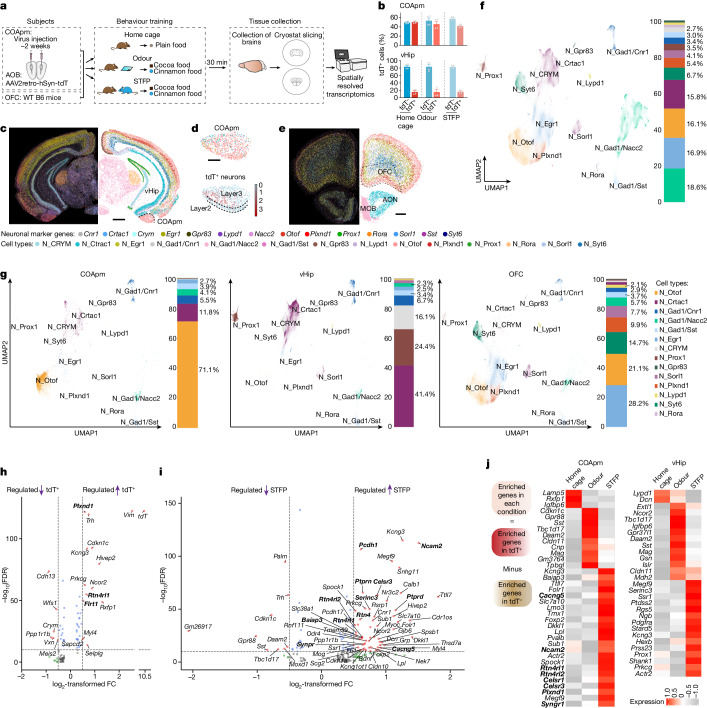
Spatially resolved transcriptomics reveals neuronal composition and STFP-training-induced changes in gene expression in the COApm, ventral hippocampus and OFC. **a**, Experimental strategy. For analyses of the COApm and the ventral hippocampus, AOB-projecting neurons were labelled by injecting the AOB with AAV2retro-hSyn-tdTomato two weeks before STFP training, whereas analyses of the OFC were performed using uninjected wild-type (WT) mice (*n* = 4 mice per group). **b**, AOB-projecting (tdT^+^) neuron density in the COApm and ventral hippocampus quantified by MERFISH (4 mice per group; mean ± s.e.m.). **c**–**e**, Spatial representations of neuronal markers and cell-type identification in brain sections containing the COApm and ventral hippocampus (**c**,**d**) or the OFC (**e**) (**c**,**e**, left, MERFISH fluorescent images (dark background); right, neuron types (white background); **d**, top, magnified COApm image (from **c**); bottom, spatial localization of tdT^+^ neurons in the COApm). Scale bars, 1 mm (**c**); 0.2 mm (**d**); 0.5 mm (**e**). **f**,**g**, Unbiased clustering of all neurons (*n* = 978,574; **f**) or separately of COApm, ventral hippocampus and OFC neurons (**g**) in a uniform manifold approximation and projection (UMAP) format with cell cluster percentages on the right. **h**,**i**, Volcano plots showing DEGs in comparisons of AOB-projecting (tdT^+^) versus AOB-nonprojecting (tdT^−^) COApm neurons in the STFP training group (**h**) or in comparisons of AOB-projecting COApm neurons in STFP training versus odour groups (**i**) (false discovery rate (FDR) < 1 × 10^−10^ by the Benjamini–Hochberg method; fold change (FC) > ±0.5). **j**, Schematic (left) and heat map of enriched genes (right) detected in excitatory AOB-projecting (tdT^+^) neurons in the COApm (left) and ventral hippocampus (right). Genes related to synapse formation are in bold.

Long-term memory formation, but not memory acquisition, requires de novo transcription of DNA and protein synthesis^45^. Consistent with such a requirement, local administration of the protein-synthesis inhibitor anisomycin^46^ into the COApm or OFC after STFP training abolished long-term STFP memory tested three weeks later (Fig. 3h,i). Thus, the essential roles of the COApm and OFC in long-term STFP memory formation are protein-synthesis dependent, raising the question of whether similar or different changes in gene expression in the COApm and OFC are involved. To address this question, we performed single-cell spatially resolved transcriptomics analyses using multiplexed error-robust fluorescence in situ hybridization (MERFISH), comparing the COApm with the OFC and the ventral hippocampus, which we included because of the ventral hippocampus’s distinct involvement in STFP memory acquisition but not long-term memory consolidation (Fig. 4a). As controls, we used home-cage mice and mice that had been exposed to cinnamon odour without a social interaction.

**Extended Data Fig. 4:**
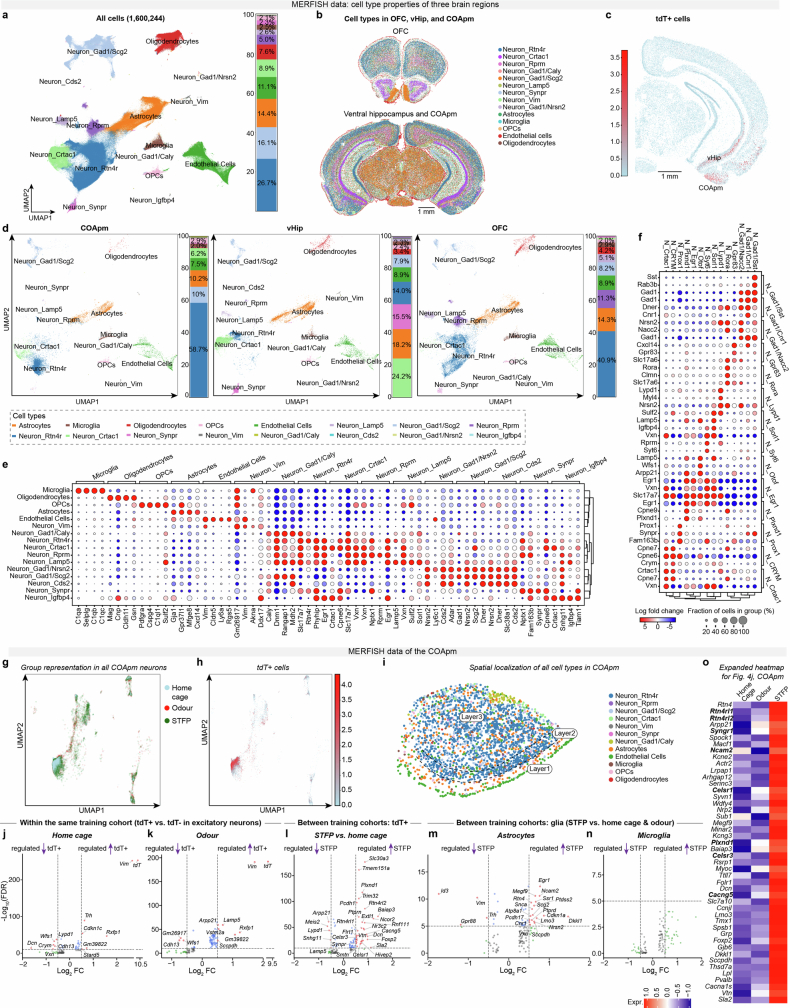
In-depth MERFISH spatially resolved transcriptomics analysis of cell types and their specific markers and of the gene-expression changes in the COApm after STFP training. **a**, Unbiased UMAP clustering of all cells identifies a total of 1.6 million cells in spatially revolved transcriptomic sections (n = 4 mice per experiment). **b**, Spatial images of all cell types in both coronal sections analysed by MERFISH. **c**, tdTomato spatial expression pattern reveals selective labelling of the COApm, ventral hippocampus, and entorhinal cortex in the coronal section containing the COApm. Note that the injection of retro-AAVs triggering tdTomato expression into the AOB is likely to involve limited spillover into the adjacent MOB and AON. As a result, the tdTomato labelling of the ventral hippocampus and entorhinal cortex could at least in part be due to MOB and/or AON projections. **d**, UMAP plots depict unbiased clustering of all cell types in the COApm, ventral hippocampus, and OFC. The bars on the right of the UMAP plots illustrate the cell composition percentages. **e**,**f**, Markers for all cell types (**e**) and all neurons (**f**) in all three brain regions as determined by MERFISH spatially resolved transcriptomics. **g**,**h**, UMAP plots showing that all cell types were consistently found in the COApm in the three experimental groups (home cage, odour, and STFP training) (**g**) but that tdT+ AOB-projecting COApm neurons are concentrated in the main Otof+ neuron type in the COApm in all three groups (see Fig. 4g, COApm, for the definition of neuron clusters). **i**, Zoomed-in MERFISH image of the COApm to illustrate cell types, with cell annotations listed on the right. **j**,**k**, Volcano plots of DEGs identified in a comparison of excitatory AOB-projecting vs. AOB-nonprojecting (tdT+ vs. tdT-) neurons of the COApm reveal no major changes in home cage (**j**) or odour (**k**) conditions in contrast to the STFP condition (see Fig. 4h). For volcano plots, dotted lines indicate an FDR<1e-10 by Benjamini–Hochberg Methodor, and a 0.5 log_2_ fold change (FC). **l**, Volcano plots of DEGs identified in a comparison of excitatory tdT+ neurons in the STFP vs. home cage conditions complementing the volcano plot for the STFP vs. odour comparison shown in Fig. 4i. FDR<1e-10, log_2_FC < 0.5. **m**,**n**, Volcano plots of DEGs identified in the comparison of STFP vs. home cage & odour conditions in astrocytes (**m**) and microglia (**n**). FDR<1e-5, log_2_FC < 0.5. **o**, Expanded heat map for the STFP-condition enriched genes of the COApm shown in Fig. 4j. Genes related to synapse formation are in bold.

We selected 336 genes (Supplementary Table 2) from single-cell RNA sequencing (scRNA-seq) data (see below) as custom probes and measured the spatial representations of neuron types in the three brain regions (Fig. 4b–e). We labelled AOB-projecting neurons in the COApm (around 50%) and ventral hippocampus (around 18%) by injecting the AOB with retrograde tdTomato, and used wild-type mice for OFC sections because the OFC does not project to the AOB^28^ (Figs. 1c and 4b and Extended Data Fig. 4c).

Unbiased clustering of more than 1.6 million cells in all sections revealed 16 cell types comprising 978,574 neurons and 5 non-neuronal cell types (Fig. 4f and Extended Data Fig. 4a–d). Cell types were largely conserved across the three brain regions, with different relative frequencies (Extended Data Fig. 4d,e). Neurons were subclustered into 14 types, revealing distinct excitatory neuron but relatively conserved inhibitory neuron cluster compositions across the three brain regions (Fig. 4g and Extended Data Fig. 4f). The cluster compositions in the COApm were similar in the three experimental groups (Extended Data Fig. 4g), suggesting that STFP training does not affect its cellular architecture.

Comparisons of differentially expressed genes (DEGs) in COApm excitatory neurons that either project (tdT^+^) or do not project (tdT^−^) to the AOB (both mainly located in clusters N_Otof and N_Crtac1; Fig. 4g,h and Extended Data Fig. 4h,i) identified significant changes in STFP-trained mice (Fig. 4h) but not in home-cage (Extended Data Fig. 4j) or odour-only mice (Extended Data Fig. 4k), consistent with the selective activation of COApm neurons by STFP training (Fig. 1g–j) and the requirement for protein synthesis in the COApm for long-term STFP memory consolidation (Fig. 3h). Robust gene-expression changes were detected in comparisons of tdT^+^ neurons between STFP-trained and home-cage or odour-only mice (Fig. 4i, Extended Data Fig. 4l and Supplementary Table 3). Notably, astrocytes also exhibited significant STFP-specific gene-expression changes, whereas microglia did not (Extended Data Fig. 4m,n).

**Extended Data Fig. 5:**
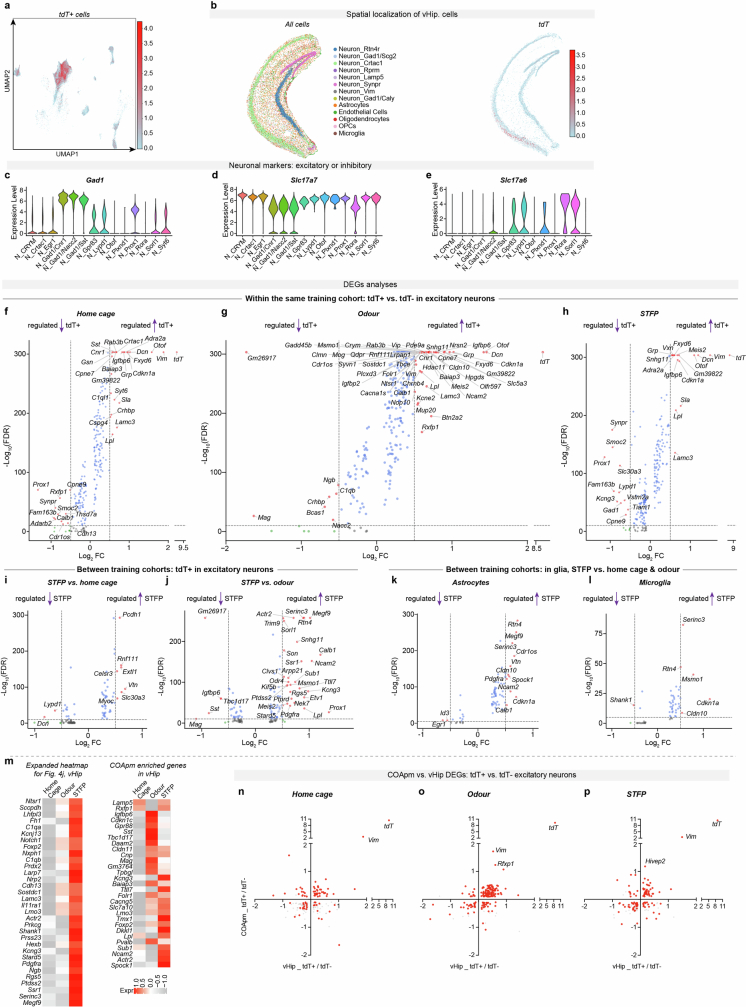
Spatially resolved transcriptomics of the ventral hippocampus reveals a unique gene-expression architecture and shows that STFP-training-induced DEGs differ between the COApm and the ventral hippocampus. **a**, UMAP plot of all tdT+ AOB-projecting neurons in the ventral hippocampus pooled from home cage, odour, and STFP training groups. **b**, Image of all cell types in the ventral hippocampus revealed by MERFISH (left) and of the spatial localization of tdT+ cells in the ventral hippocampus (right, red on blue). **c**–**e**, Expression levels of three neurotransmitter markers (**c**, Gad1 [GABA synthesis]; **d**, Slc17a7 [vesicular glutamate transporter vGluT1]; **e**, Slc17a6 [vesicular glutamate transporter vGluT2]) in the 14 types of neurons in MERFISH analyses of the ventral hippocampus of the pooled groups. **f**–**h**, Volcano plots uncovering DEGs in a comparison of tdT+ vs. tdT- excitatory neurons of the ventral hippocampus in home cage (**f**), odour (**g**), or STFP-trained mouse groups (**h**). For volcano plots, dotted lines indicate an FDR <1e-10 by Benjamini–Hochberg Method and a 0.5 log_2_ fold change (FC). **i**,**j**, Volcano plots showing DEGs in a comparison of tdT+ excitatory neurons between STFP vs. home cage (**i**), and STFP vs. odour mouse groups (**j**). FDR<1e-10, log_2_(FC) < 0.5. **k**,**l**, Volcano plots revealing DEGs in a comparison of STFP vs. home cage & odour in astrocytes (**k**) and microglia (**l**). FDR<1e-5, log_2_(FC) < 0.5. **m**, Heat maps of enriched genes in the ventral hippocampus. The left heat map shows an expanded analysis of enriched genes in the ventral hippocampus corresponding to the right panel of Fig. 4j, while the right heat map shows the expression of COApm-enriched genes (from the left panel of Fig. 4j) in the ventral hippocampus. **n**–**p**, Scatter plots comparing DEGs identified tdT+ vs. tdT- neurons in the COApm and the ventral hippocampus under home cage (**n**), odour (**o**) or STFP (**p**) conditions.

We next analysed the changes in gene expression in the ventral hippocampus, which also projects to the AOB^28,47^ (Figs. 1c and Fig. 4b and Extended Data Fig. 4c). Most tdT^+^ neurons in the ventral hippocampus are *Crtac1*^+^ neurons located in the ventral CA1 subdivision (Extended Data Fig. 5a–e). Comparisons of DEGs between tdT^+^ and tdT^−^ excitatory neurons within a training cohort suggested more extensive changes in the odour group (Extended Data Fig. 5g) than in the home-cage or STFP groups (Extended Data Fig. 5f,h). Moreover, a comparison of DEGs in tdT^+^ excitatory neurons across the three groups uncovered more robust changes in STFP versus odour than in STFP versus home cage or in glia comparisons (Extended Data Fig. 5i–l). Thus, gene-expression changes in the ventral hippocampus are induced mainly by odour perception.

**Extended Data Fig. 6:**
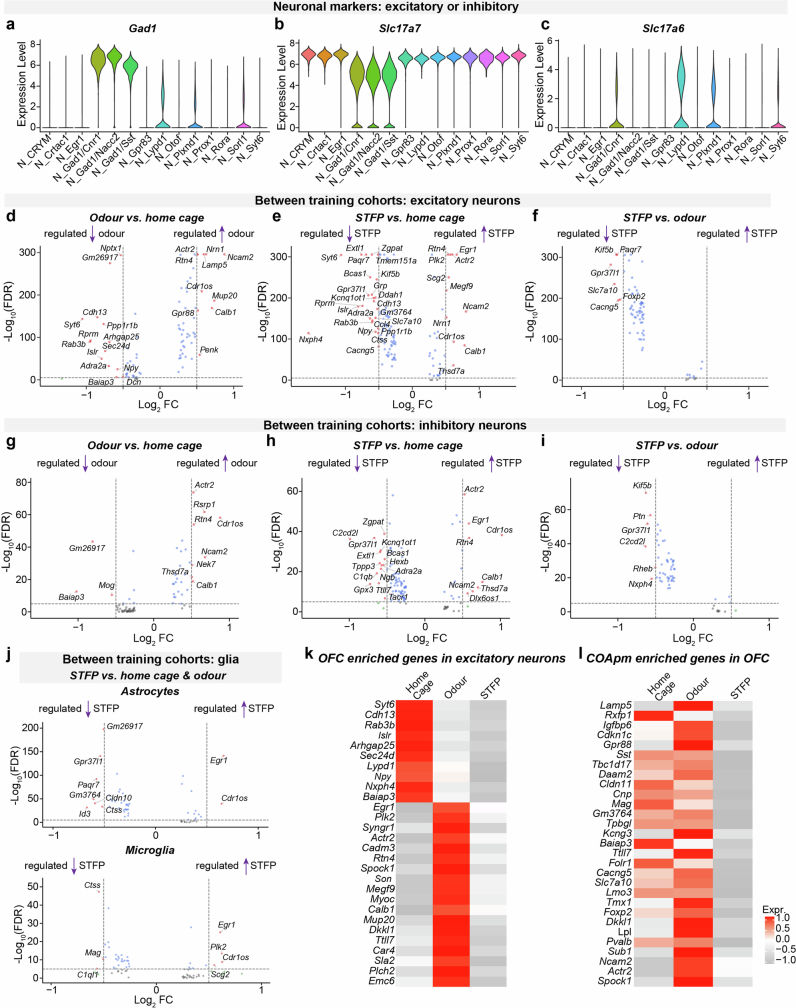
Spatially resolved transcriptomics identifies major changes in gene expression in the OFC that are driven mainly by odour perception even though the OFC does not receive direct inputs from the olfactory bulb. **a**–**c**, Expression levels of three neurotransmitter markers (**a**, Gad1 [GABA synthesis]; **b**, Slc17a7 [vesicular glutamate transporter vGluT1]; **c**, Slc17a6 [vesicular glutamate transporter vGluT2]) in the 14 types of neurons identified in MERFISH spatially resolved transcriptomic analyses of the OFC in the pooled home cage, odour, and STFP-trained groups. **d**–**f**, Volcano plots analysing gene-expression changes in excitatory neurons by comparing odour vs. home cage (**d**), STFP vs. home cage (**e**), and STFP vs. odour (**f**). For these and the following volcano plots, dotted lines indicate an FDR <1e-5 by Benjamini–Hochberg Method and a 0.5 log_2_ fold change (FC). **g**–**i**, Same as **d**–**f** but in inhibitory neurons. **j**, Volcano plots analysing gene-expression changes in astrocytes (top) and microglia (bottom) by comparing STFP training conditions with home cage and odour conditions. **k**, Heat map of OFC enriched genes in excitatory neurons. **l**, Heat map illustrating the expression of COApm-enriched genes (same genes as in the left panel of Fig. 4j) in the OFC.

Finally, we examined changes in gene expression in the OFC, which is required for STFP long-term memory formation over a broad time window^6,10,11^ (Fig. 2g and Extended Data Fig. 2x). DEG computations in excitatory and inhibitory neurons between the three behavioural conditions uncovered a trend towards gene-expression changes induced by odour and maintained by STFP training (Extended Data Fig. 6a–i). Heat maps of OFC STFP-enriched genes (Extended Data Fig. 6k) and of COApm STFP-training-induced genes in the OFC (Extended Data Fig. 6l) again revealed a gene signature dominated by odour exposure instead of by STFP training. Moreover, unlike the COApm, the OFC did not show significant gene-expression changes in the MERFISH spatially resolved transcriptome of astrocytes and microglia (Extended Data Fig. 6j).

The finding that odour- but not STFP-training-induced DEGs dominate in the ventral hippocampus and OFC indicates that the STFP-training-induced gene-expression programs probably differ between the COApm, ventral hippocampus and OFC, as we confirmed in a direct analysis (Fig. 4j and Extended Data Figs. 4o, 5m–p and 6k,l). Gene-expression changes after odour stimulation, by contrast, are more consistent (Extended Data Fig. 5m,o). Overall, these results suggest that gene-expression signatures in the COApm are selectively activated by STFP training and differ from those of the OFC and ventral hippocampus, which are often activated by odour stimulation alone.

## STFP memory consolidation genes

**Fig. 5:**
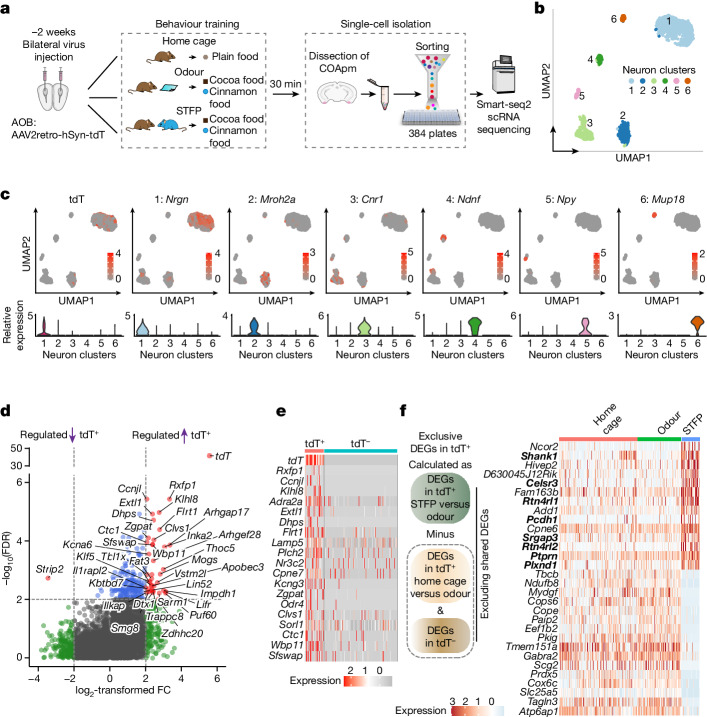
Deep scRNA-seq reveals that STFP training induces marked changes in gene expression in COApm neurons. **a**, Experimental strategy. **b**, Unbiased clustering shown in UMAP plots of scRNA-seq transcriptomes of COApm neurons. **c**, COApm neuron subtypes are identified by distinct marker genes, with expression of tdTomato highly enriched in cluster 1. **d**, Volcano plots showing DEGs detected in the STFP-trained mouse group in comparisons of AOB-projecting (tdT^+^) and nonprojecting (tdT^−^) excitatory neurons (cluster 1) of the COApm (FDR < 1 × 10^−2^ by the Benjamini–Hochberg method). **e**, Top upregulated DEGs (ranked by *P* value) in AOB-projecting (tdT^+^) neurons. **f**, STFP memory-specific DEGs. Left, computation strategy; right, heat map of identified STFP-specific DEGs in the home cage, odour and STFP-training groups (see Supplementary Table 4 for details). Genes related to synapse formation are in bold.

We next investigated which gene-expression changes in the COApm inform its unique function in memory consolidation. We applied the same experimental design that was used in the MERFISH spatially resolved transcriptomics experiments to full-length scRNA-seq experiments using a Smart-seq2 protocol (Fig. 5a), which enabled an average sequencing depth of 1.5 million reads per cell.

**Extended Data Fig. 7:**
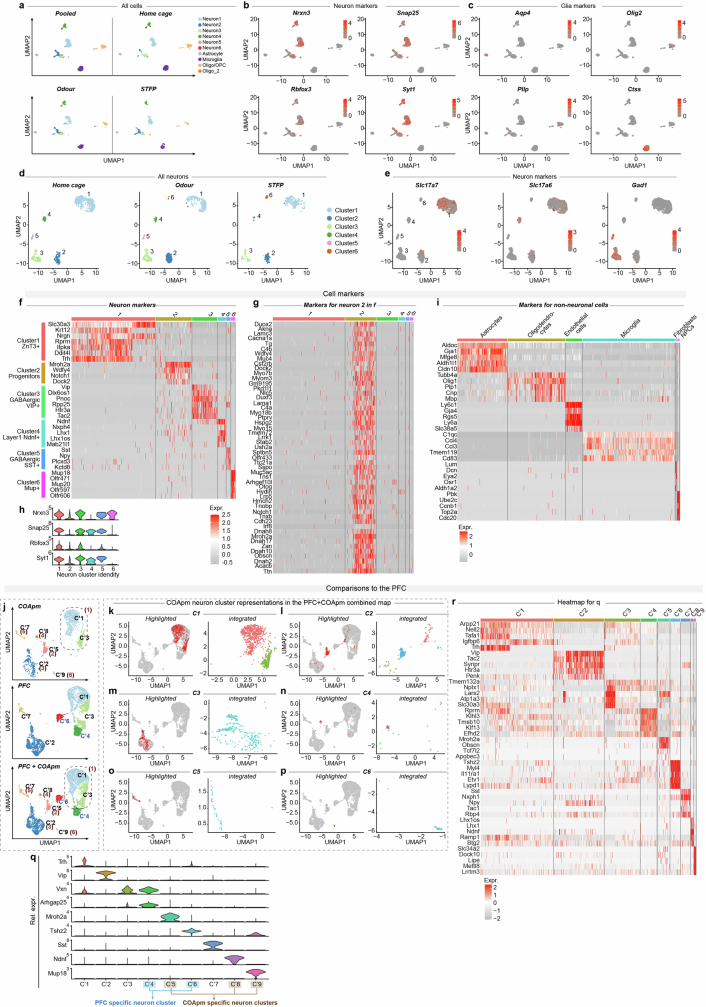
scRNA-seq reveals a unique cellular composition of the COApm that only partly overlaps with that of the PFC neurons, as shown by an integrated analysis. **a**, In-depth scRNA-seq of COApm identified six major cell types that are similarly present in all three behavioural conditions analysed (home cage, odour only, and STFP training). **b**, The expression of *Nrxn3*, *Snap25*, *Rbfox3*, and *Syt1* is enriched in all neuron clusters. **c**, The glia marker *Aqp4* is expressed in astrocytes, *Oligo2* and *Pllp* in OPCs, and *Ctss* in microglia. **d**, Subclustering of COApm neurons reveals six principal neuronal cell types that are similarly abundant in all three conditions. **e**, Expression of the excitatory neuron markers *Slc17a7* and *Slc17a6* is enriched in clusters 1, 2, and 4, whereas expression of the inhibitory neuron marker *Gad1* is enriched in clusters 3 and 5. **f**, Heat map of the distinct marker genes of each neuronal cell type in the COApm. **g**, Heat map illustrating a specific gene cluster that is selectively enriched in the unusual progenitor-like neuron cluster 2 in the COApm, a neuron type that was not previously identified. **h**, Violin plots showing that one or more neuronal marker genes (*Nrxn3*, *Snap25*, *Rbfox3*, *Syt1*) are expressed in the six neuron clusters of the COApm. **i**, Integrated analysis of PFC and COApm non-neuronal cells reveals six cell clusters. **j**–**p**, Integrated analysis of neuronal transcriptomes of the COApm and the prefrontal cortex (PFC)^48^ reveals nine clusters corresponding to neuronal cell types C’1-C’9 (I.C’1-I.C’9). Four of these neuronal cell types are found in both the COApm and PFC (I.C’1-I.C’3, I.C’7) (**j**). As a cross preference, C1 cells from COApm were distributed in I.C’1 and I.C’3 (**k**), C3 cells in I.C’2 (**m**), and C5 in I.C’7 (**o**). Three clusters were more abundant in the COApm than the PFC, including I.C’5 (Mroh2a^+^, corresponding to C2) (**m**), I.C’8 (Ndnf^+^, C4) (**n**), I.C’9 (Mup18, C6) (**p**). Two neuron types, I.C’4 (Arhgap25^+^) and I.C’6 (Tshz2^+^) were enriched in PFC. **q**,**r**, Expression levels of distinct cell markers for the nine types of neurons are shown in a violin plot (**q**) and heat map (**r**).

Unbiased classifications identified 1,694 neurons (Fig. 5b and Extended Data Fig. 7b) and 1,621 non-neuronal cells in four clusters (Extended Data Fig. 7c). All cell types were consistently found in the three experimental groups (Extended Data Fig. 7a,d). Subclustering revealed six neuron clusters (COA1–COA6) comprising glutamatergic (clusters 1, 2 and 4) and GABAergic neurons (clusters 3 and 5) (Fig. 5b,c and Extended Data Fig. 7e,f). Most AOB-projecting COApm neurons, identified by tdTomato expression, were found in cluster 1 (Fig. 5c). Clusters 2 and 6 are markedly different from previously described cortical neurons. Cluster 2 neurons express high levels of *Mroh2a*, which encodes an intracellular HEAT domain protein, and co-express neuronal stem cell markers (*Notch1*, *Nestin* and *Cdk1*) with mature neuronal markers (Extended Data Fig. 7g,h). Cluster 6 neurons contain high levels of mRNAs that encode olfactory receptors (*Olfr471*, *Olfr597* and *Olfr606*, also known as *Or5p5c-ps1*, *Or52ab2* and *Or51l14*, respectively) and pheromone-binding proteins (*Mup18* and *Mup20*) (Extended Data Fig. 7f,h), suggesting that they are related to olfactory information transduction and pheromone signalling.

Integrated analysis of the transcriptomes of the COApm and the PFC^48^, another cortical area for which deep scRNA-seq data are available, revealed nine neuronal cell types that only partly overlapped (Extended Data Fig. 7j–r), whereas their glia cell types were nearly identical (Extended Data Fig. 7i). Thus, the COApm and PFC are notably different, consistent with their distinct functions (Supplementary Discussion section 2).

**Extended Data Fig. 8:**
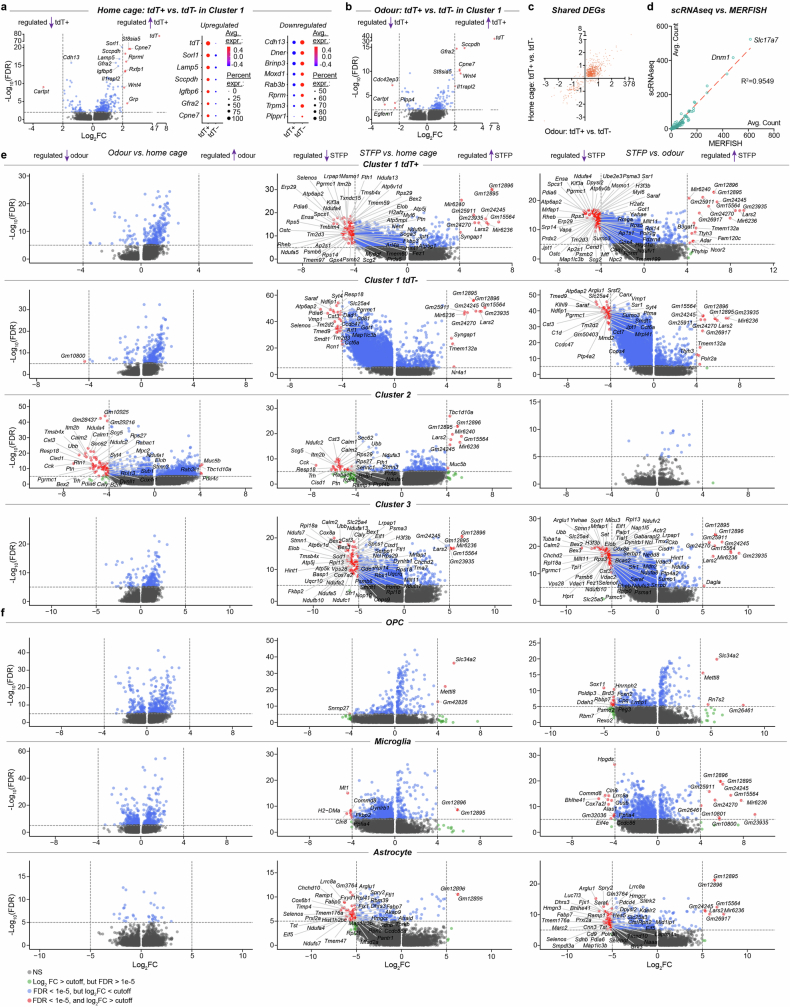
Differential gene expression in different neuron subtypes and glial cell types of the COApm compared between home cage, odour and STFP-trained mice uncovers widespread transcriptome changes in three types of neurons and in astrocytes. **a**–**c**, Comparison of the gene-expression signature of cluster 1 neurons that project (tdT^+^) or do not project (tdT-) to the AOB under the home cage (**a**) or odour (**b**) conditions, and correlation of the DEGs under these two conditions (**c**). **d**, Correlation analysis of MERFISH and scRNA-seq datasets reveals excellent correspondence between the two methods (*R*^2^ was calculated by the linear regression model). **e**, Volcano plots analysing gene-expression changes induced by the three behavioural conditions (home cage, odour, and STFP training) for cluster 1, 2 and 3 neurons uncover widespread STFP-specific changes in AOB-projecting (tdT^+^) and non-AOB-projecting neurons (tdT-) of cluster 1 and in the neurons of cluster 3, but only few STFP-specific changes in cluster 2. **f**, Volcano plots analysing gene-expression changes induced by the three behavioural conditions (home cage, odour, and STFP training) in OPCs, microglia, and astrocytes identify major STFP-specific changes only in astrocytes but not in OPCs or microglia. For all volcano plots, dotted lines indicate an FDR<1e-5 by the Benjamini–Hochberg method and a log_2_ fold change (FC) of 4 or 5.

To assess STFP-induced transcriptional changes, we compared the transcriptomes of cluster 1 COApm neurons that either project (tdT^+^) or do not project (tdT^−^) to the AOB (Fig. 5d,e and Extended Data Fig. 8a–c). In the home cage and odour-only conditions, only a small number of genes were selectively enriched (*Sorl1*, *Cpne7* and *Lamp5*) or de-enriched (*Cdh13* and *Cartpt*) in tdT^+^ neurons, with no major differences between the two conditions (Extended Data Fig. 8a–c). Thus, odour itself did not significantly affect COApm transcription. Of note, STFP training induced marked transcriptional changes in tdT^+^ versus tdT^−^ neurons of cluster 1 (Fig. 5d,e), including genes that are related to synapse formation (for example, *Flrt1*). Pairwise comparisons confirmed that STFP training stimulated significantly more transcriptional changes than did odour stimulation in COApm neurons (Extended Data Fig. 8e), consistent with the MERFISH spatially resolved transcriptomics results.

To further characterize the STFP-induced DEGs in AOB-projecting COApm neurons, we identified exclusive DEGs by removing DEGs that are also present in tdT^−^ neurons or in odour and home-cage conditions, resulting in a set of ‘STFP-specific DEGs’ (Fig. 5f and Supplementary Table 4). Notably, the top 15 most upregulated genes included a strong enrichment of synaptic cell-adhesion molecules (*Celsr3*, *Rtn4rl1*, *Rtn4rl2*, *Plxnd1*, *Ptprn* and *Pcdh1*) and transcription factors (*Ncor2* and *Hivep2*) (Fig. 5f). The changes in the expression of synaptic cell-adhesion molecules align well with the MERFISH spatially resolved transcriptomics findings (Fig. 4 and Extended Data Fig. 4o), which suggests that synapse restructuring is a central component of STFP memory consolidation.

Besides neurons, astrocytes exhibited substantial gene-expression changes induced by STFP memory formation but not by odour stimulation (Extended Data Fig. 8f), suggesting that astrocytes are actively involved in STFP memory consolidation, consistent with the MERFISH data. Thus, our data corroborate the notion that memory consolidation does not simply consist of signal integration in neurons but includes transcriptional remodelling of the overall neuronal state accompanied by related changes in surrounding glia^48,49^.

## The COApm–AON circuit consolidates memory

**Fig. 6:**
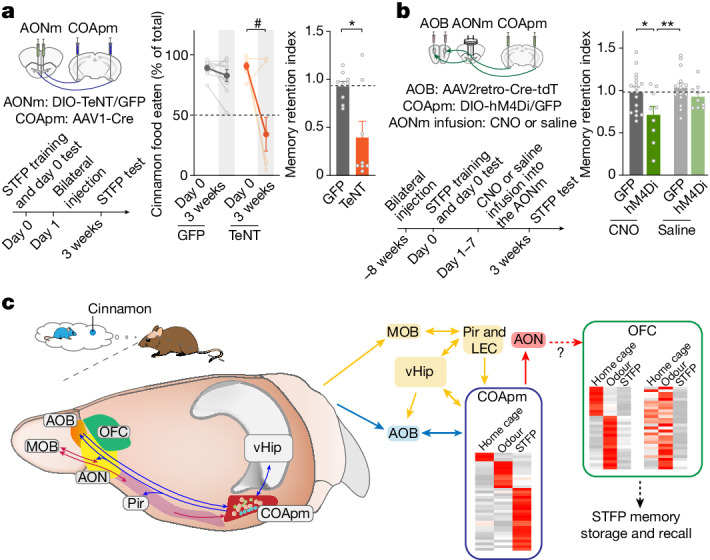
Projections from the COApm to the AONm mediate the transfer of memories consolidated in the COApm. **a**, TeNT silencing of AONm neurons that receive synaptic inputs from the COApm, one day after STFP training. Left, experimental approach; middle, percentage of cinnamon-flavoured food eaten on day 0 and after 3 weeks (*P* = 0.0234, two-tailed Wilcoxon signed-rank test); right, memory retention indices (*P* = 0.0464, two-tailed Mann–Whitney test) (with GFP, *n* = 9; TeNT, *n* = 8 mice). **b**, Chemogenetic inhibition of COApm projections to the AONm during the first week after STFP training impairs STFP memory (CNO-GFP, *n* = 17; CNO-hM4Di, *n* = 9; saline-GFP, *n* = 15, saline-hM4Di, *n* = 9; *F*_3,46_ = 3.995, *P* = 0.0131, one-way ANOVA with Tukey post-hoc test). **c**, Summary diagram of the central role of the COApm and its interactions with various brain regions in STFP memory consolidation. Left, schematic of brain circuits; right, information flow during STFP consolidation. LEC, lateral entorhinal cortex. Pir, piriform cortex. Brain map based on reference coordinates from the Allen Mouse Brain Reference Map, Allen Institute for Brain Science (http://atlas.brain-map.org/). Data in **a**,**b** are mean ± s.e.m. For details of statistics, see Supplementary Tables 5 and 6. #, *P < 0.05, **P < 0.01. Source Data

**Extended Data Fig. 9:**
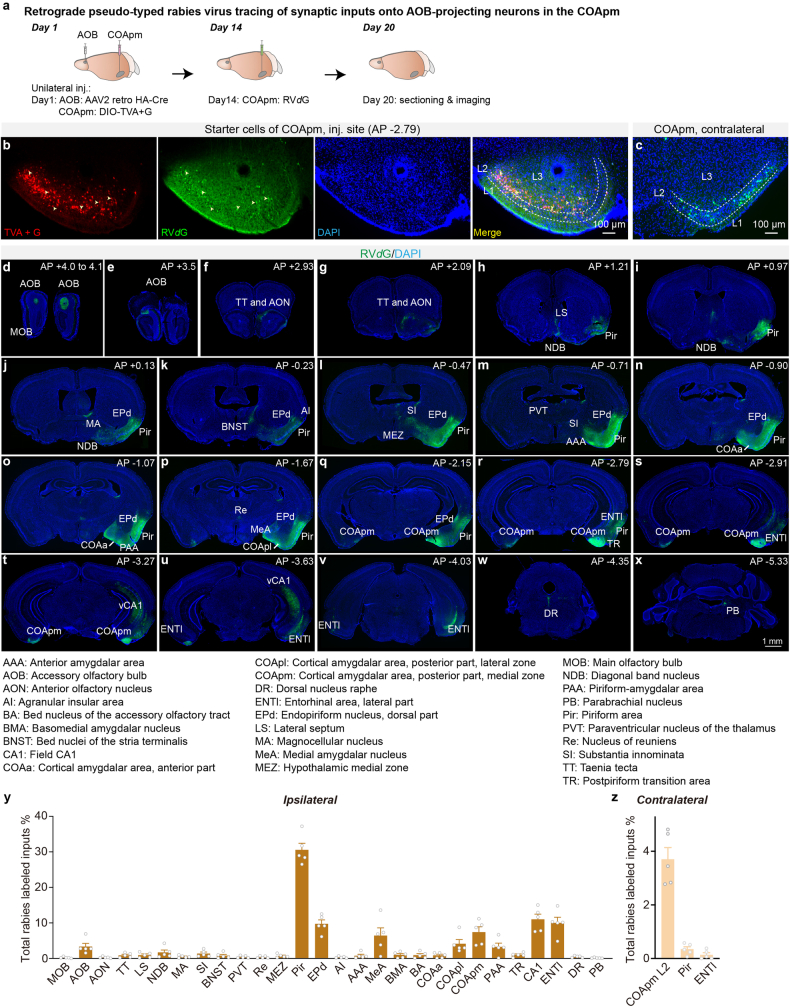
Retrograde pseudotyped rabies virus tracing of AOB-projecting COApm neurons. **a**, Experimental strategy. **b**, Representative images of starter cells in injection sites, with tdT+ starter cells shown in red. Note that a large fraction of the green GFP+ cells do not overlap with the red starter cells, demonstrating that the COApm contains local synaptic networks. **c**, Representative image of the contralateral COApm illustrating that a subset of neurons in layer 2 forms synaptic inputs onto the contralateral COApm. **d**–**x**, Representative coronal sections of brain slices showing retrograde pseudo-rabies virus-labelled inputs into COApm AOB-projecting neurons (green = GFP). Abbreviations designating brain regions are explained below the images, and the positions of the sections are listed in the top right corner of every image. Scale bar in the last image applies to all images in a set. **y**,**z**, Quantification of the relative number of synaptic inputs onto AOB-projecting neurons in the COApm from other regions of the entire mouse brain from images acquired from 5 mice. (**y**) ipsilateral; (**z**) contralateral. Note that identified multiple ipsilateral brain regions, including the AOB, provide inputs into COApm neurons, consistent with our optogenetic recording results (Fig. 1d–f and Extended Data Fig. 1p,q). In contrast, few contralateral brain regions provide synaptic inputs, including contralateral layer-2 COApm neurons. The results agree with retrograde tracing data obtained using Fluorogold^29^, but extend these data in identifying AOB-projecting COApm neurons as targets. Data are means ± s.e.m. (n = 5 mice). Source Data

**Extended Data Fig. 10:**
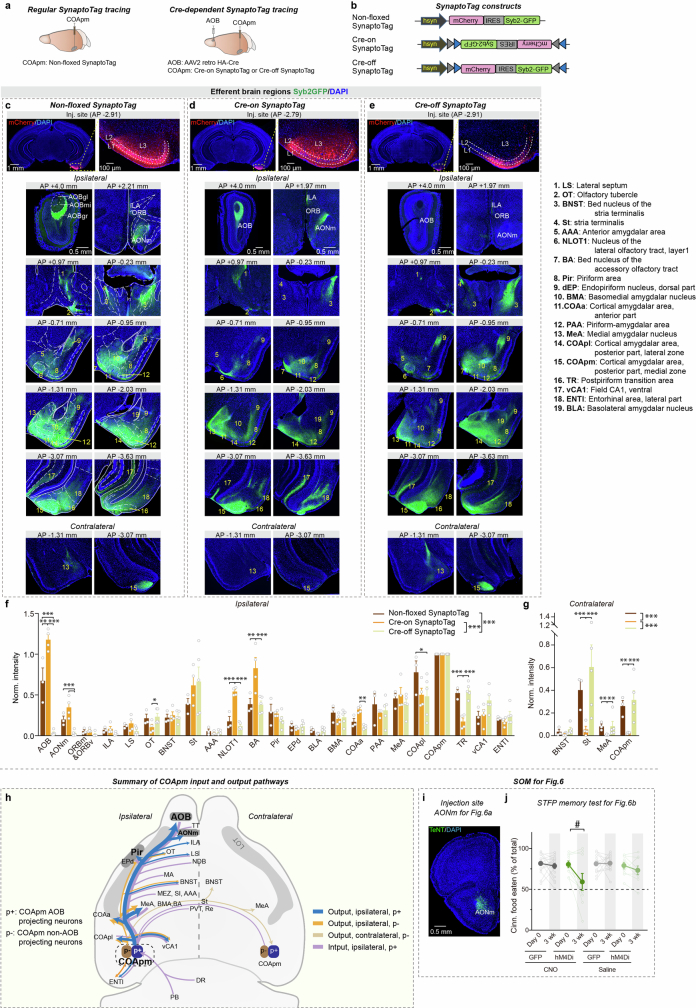
Brain-wide mapping of presynaptic projections by COApm neurons to target brain regions using SynaptoTag tracing. **a**, Experimental strategy. Three different types of SynaptoTag mapping were performed: mapping all projections from the COApm by standard SynaptoTag^54^; mapping COApm AOB-projecting neurons using Cre-dependent Cre-on SynaptoTag^33^; and mapping only the COApm non-AOB-projecting neurons using Cre-off SynaptoTag. **b**, Maps of SynaptoTag constructs that co-express mCherry as a cytoplasmic marker allowing tracing of axons with GFP-tagged synaptobrevin-2 as a presynaptic terminal marker. **c**–**e**, Representative images of SynaptoTag mapping experiments analysing projections of all COApm neurons (**c**), of only AOB-projecting neurons (**d**), or of only non-AOB-projecting neurons (**e**). Injection site images of the COApm are shown on top, and images from different target regions are shown below, with the regions identified by numbers that are explained on the right of the images. Scale bars apply to all images in a set in **c** and **e**. In **d**, scale bar in the AP+1.97 image applies to the rest. **f**,**g**, Quantifications of target projections of COApm neurons obtained with the three different SynaptoTag strategies described above, with the intensity of SynaptoTag staining normalized to the injection site signal in the COApm. Regular SynaptoTag, n = 3; Cre-on SynaptoTag, n = 4; Cre-off SynaptoTag, n = 5, mice. For **f**, F_2, 207_ = 20.74, p = 6.2 × 10^−9^; for **g**, F_2, 36_ = 31.61, p = 1.2 × 10^−8^. **h**, Summary of the inputs and output maps of the COApm as determined by pseudo-rabies virus and SynaptoTag tracing experiments. **i**,**j**. Supplementary data for Fig. 6. **i**, representative image of TeNT expression in the AONm. **j**, percentage of cinnamon-flavoured food consumed during day 0 and 3-week food-choice test for Fig. 6b, CNO-GFP, n = 17; CNO-hM4Di, n = 9; saline-GFP, n = 15, saline-hM4Di, n = 9. CNO, hM4Di, 3 wk vs. day 0, t_8_ = 2.739, p = 0.0255. All data are means ± s.e.m. Two-way ANOVA with post-hoc Tukey test was used to detect differences in the SynaptoTag tracings. Data are transformed by taking square root (**f**) or Ln (**g**) first to make sure data are normally distributed and have equal variances. *p < 0.05, **p < 0.01, ***p < 0.001. Paired two-tailed student *t*-test was applied to (**j**), with #*<0.05, **<0.01, ***<0.001. For details, see Supplementary Tables 5 and 6. Source Data

To gain insight into how a memory that was consolidated in the COApm is subsequently computationally processed and stored, we mapped synaptic input and output connections of the COApm using retrograde pseudotyped rabies virus^50^ and SynaptoTag tools^33^. We analysed both AOB-projecting and AOB-nonprojecting COApm neurons (Extended Data Figs. 9 and 10). Consistent with previous reports^27,29^, we found that COApm neurons form reciprocal connections with olfactory cortices, the ventral hippocampus and various amygdalar areas (Extended Data Figs. 9 and 10). Notably, the piriform cortex emerged as a major source of COApm inputs, supporting the notion that the COApm integrates contextual olfactory sensory and social cues (Extended Data Fig. 9). Moreover, the medial nucleus of the anterior olfactory nucleus (AONm), which is known to contribute to STFP memory formation^13^, is among the foremost projection targets of AOB-projecting COApm neurons. Combining the circuit and transcriptomics data, we thus hypothesized that STFP-related social information is transferred from the AOB to the COApm, where it is integrated with sensory odour information from the ventral hippocampus and the piriform cortex and then transmitted via the AONm to higher-order cortices for memory storage (Fig. 6c).

To test this hypothesis, we inactivated AONm neurons that receive COApm inputs by infecting the COApm with AAV1-Cre and the AONm with AAV-DJs DIO-TeNT or GFP (Fig. 6a). Silencing of AONm neurons after STFP training disrupted long-term STFP memory, indicating that the AONm is essential for the transmission of memory consolidation signals from the COApm (Fig. 6a and Extended Data Fig. 10i).

To independently confirm this conclusion, we again used chemogenetics (Fig. 6b). We expressed hM4Di in COApm AOB-projecting neurons that also project to the AONm. After training, we infused CNO locally into the AONm via stereotactic manipulations, with GFP and saline controls (Extended Data Fig. 10j). Inhibiting the COApm output to the AONm again selectively impaired long-term STFP memory (Fig. 6b and Extended Data Fig. 10j), validating the conclusion that COApm–AONm projections communicate STFP memory consolidation. Note that we found that a similar experiment for the AOB does not decrease memory consolidation (Extended Data Fig. 3h), which serves as an additional control for the AONm manipulations.

## Summary

Here we show that STFP memory is rapidly consolidated in a defined cortical nucleus, the COApm, whose selective role in STFP memory formation seems to be to mediate protein-synthesis-dependent computations that synthesize social and olfactory inputs. We propose that social inputs are transmitted directly from the AOB to the COApm, whereas olfactory inputs are transmitted indirectly from the MOB via the piriform cortex and ventral hippocampus. We show that approximately half of COApm neurons form synaptic connections with the AOB and AON that are selectively essential for memory consolidation but not for short-term memory acquisition or long-term memory storage and recall. By contrast, the AOB is only required during STFP training, whereas the AON has multiple roles in STFP. The selective function of COApm neurons in long-term STFP memory consolidation differs from that of other brain regions tested, and involves major changes in gene expression as analysed by deep scRNA-seq and spatially resolved transcriptomics. These gene-expression changes are unique to the COApm when compared to the ventral hippocampus and OFC, both of which contribute to STFP memory formation. The COApm might perform further behavioural functions in mice that, given its dense direct AOB and indirect MOB inputs, are also likely to involve an integration of social and olfactory information, such as that which occurs during mating^4^. Thus, we propose that the COApm functions as a computational memory consolidation centre. This suggests that long-term memory formation can be deconstructed into several protein-synthesis-dependent phases that are localized to distinct neural circuits, which, at least in the case of the COApm circuit, can involve a restructuring of synapses (Fig. 6c).

## Methods

### Animal procedures

C57BL/6J (Jax stock: 000664), Ai75 (Jax stock: 025106), Ai14 (Jax stock: 007914), Sun1-sfGFP (Jax stock: 030952)^51^, vGAT-Cre (Jax stock: 028862), PV-Cre (Jax stock: 008069), vGluT2-Cre (Jax stock: 028863), SST-Cre (Jax stock: 013044) and CAMKII-Cre (Jax stock: 005359) mice were purchased from The Jackson Laboratory and bred in house. Genotyping for each line was performed using primers recommended by The Jackson Laboratory (https://www.jax.org/). TRAP2 mice^31^ (a gift from L. Luo’s laboratory, also Jax stock: 030323) were crossed for specific experiments with Ai75 or C57BL/6J mice. Only male mice were used for experiments, and all mouse lines were maintained on a C57BL/6J background. Heterozygotes for *Fos*^*2A-iCreER*^ and Ai75 alleles were used in behaviour tests. Mice were ordered from The Jackson Laboratory, and acclimated at the Stanford animal facility for at least two weeks. Mice were fed ad libitum on the ENVIGO (T2918.15) diet throughout the study. Mice were housed in groups with up to five mice per cage and on 12-h light–dark cycles (07:00–19:00, light) before behaviour experiments. After STFP training, test mice were single-housed until food-choice tests were done. All behaviour experiments were performed during the same circadian period by experimenters unaware of the subject identity. All protocols and husbandry conditions were approved by the Administrative Panel on Laboratory Animal Care at Stanford University under the guidelines of the National Institutes of Health for the care and use of laboratory animals.

### Pharmacological agents

Tamoxifen (Sigma, T5648) stock solutions were prepared by dissolving tamoxifen in corn oil (Sigma, C8267) in the presence of ethanol, which was then evaporated before use in a speed vac as described^52^. Tamoxifen was administered intraperitoneally once daily at 150 mg per kg from day 1 to day 5 after STFP training. CNO (Tocris, 4936) or saline vehicle was administered intraperitoneally at 2 mg per kg twice daily or 40 min before the food-choice test. For experiments in which CNO was injected during the entire three weeks or only during the third week, CNO injections were stopped 24–48 h before the three-week food-choice test. For the CNO terminal infusion into the AONm, 200 nl CNO at 2.5 pg nl^−1^ was delivered bilaterally through an infuser connected to a microinfusion pump (WPI, SP101I), which was left in place for an additional 2 min to allow the drug to fully diffuse before extraction. CNO or vehicle saline was microinfused twice daily from day 1 to day 7 after STFP training for the experiments in Fig. 6b. Anisomycin (Sigma A9789) was prepared as described^53^ and infused into the OFC through an infuser or stereotactically injected into the COApm immediately after STFP training.

### Plasmid construction and AAV preparations

AAV-DO_DIO (Addgene 37120), TeNT, non-floxed SynaptoTag and Cre-on SynaptoTag constructs were described previously^33,54^^,^^55^. For Cre-off SynaptoTag and Cre-off TeNT, the elements between the two loxPs were flipped^55^. For HA-Cre, the GFP moiety of Cre-GFP was replaced with an HA tag. Plasmids were converted into adeno-associated viruses (AAVs) with the AAV-DJ^56^, AAV2retro^57^ or AAV1(AAV1-Cre)^58^ serotype. In brief, helper plasmid (phelper) and capsid plasmids (pDJ or AAV2retro) were co-transfected with virus plasmid into HEK293T cells (ATCC, CRL-11268) using calcium phosphate. Then, 72 h after transfection, cells were collected and lysed, and the supernatant was loaded onto an iodixanol gradient medium (Accurate, AN1114542) and ultracentrifuged at 65,000 rpm at 4 °C for 3 h. AAVs were then extracted from the 40% iodixanol layer, washed, concentrated, aliquoted and stored at −80 °C until use. hSyn-DIO-hM4Di-IRES-GFP AAVs were a gift from X. Chen’s laboratory at Stanford University.

### Stereotactic injections and cannula implantation

Eight-week-old mice were anaesthetized with 250 mg per kg tribromoethanol (Sigma, T48402). Carprofen (5 mg per kg) was injected subcutaneously before and after surgery as an anti-analgesic. The following coordinates were used (AP, anterior to bregma; ML, lateral to midline; DV, ventral to dura; in mm): (1) COApm, AP −2.80, ML ±2.85, DV −5.1; (2) AONm, AP +2.33, ML ±0.5, DV −3.0; (3) mPFC, AP +2.0, ML ±0.3, DV −2.0; (4) OFC, AP +2.7, ML ±1.2, DV −1.8; (5) BLA, AP −1.4, ML ±3.4, DV −4.5; (6) ventral hippocampus, two sets of coordinates were used, AP −3.3, ML ±3.2, DV −3.2 and −2.0; AP −3.3, ML ±2.5, DV −3.6 and −1.8. For the AOB, AP was recognized by both the distance from bregma +4.0 mm and posterior to the inferior cerebral vein, ML ± 0.88, DV −0.88. Before injecting the AOB, the skull was adjusted at an angle of around 30°, which made the bregma higher than the lambda, and surgeries were carefully conducted to avoid damaging the inferior cerebral vein. Viruses were injected through a beveled glass pipette connected to a nanolitre injector (WPI, NL2010MC2T) at a rate of 0.1–0.25 μl per min. Injection started 1 min after the glass pipette had reached the DV depth, and the glass pipette was removed slowly 10 min after the injection was done.

For AONm drug infusions, the bilateral guide cannula (2.1 mm in length, 1.2 mm centre to centre) was implanted above the AONm and used with an infuser (33 gauge, 1.0 mm projection). For AOB drug infusions, the bilateral guide cannula (0.88 mm in length, 2 mm centre to centre) was implanted above the AOB and used again with an infuser (0.5 mm projection). For OFC infusions, the bilateral guide cannula (1.3 mm in length, 2.2 mm centre to centre) was implanted above the OFC and also used with an infuser (0.5 mm projection). Because the implantation of a cannula above the COApm will damage the ventral hippocampus that is also essential for the STFP memory acquisition, we stereotactically injected anisomycin into the COApm immediately after STFP training.

Biocytin labelling to map local dendrites of neurons was performed by patching neurons and filling them with biocytin, followed by imaging. Neurons were identified after labelling them with two approaches, infection of the AOB of C57BL/6J mice with a mixture of AAV expressing anterograde EYFP and AAV2retro-hSyn-tdTomato viruses, or infection of Sun1-sfGFP mice with a mixture of AAV expressing anterograde mCherry and AAV2retro-hSyn-Cre-HA viruses.

The intervals between virus injections and analyses are stated in the figures, except for the SynaptoTag tracing experiments, in which eight-week-old C57/BL6J mice were injected with viruses and analysed six to eight weeks afterwards.

### Behavioural tests

#### Production of flavoured food pellets and innate food-preference tests

The production of flavoured food pellets and the innate food-preference tests were performed as described^7,13^. In brief, scented food pellets were made using food powders produced in a blender from normal mouse chow (ENVIGO, T2918.15). Food powders were mixed with ground cinnamon (McCormick; final concentration of 1%), cocoa powder (Hershey’s, 100%, non-sweetened; final concentration of 2%^59^), ground cumin (McCormick; final concentration of 0.5%) or ground thyme (McCormick; final concentration of 0.75%^38^). For innate food-preference tests, mice naive to the odours used were fasted for 15–18 h and then given two food choices (cocoa versus cinnamon, or cumin versus thyme) for one hour. The food pellet was weighed before and after the test. The proportion of each flavoured food consumed was calculated as the ratio to the total food consumed. In all figures, cinnamon-flavoured food is represented by a solid circle, cocoa-flavoured food by a solid square, cumin-flavoured food by a hollow circle and thyme-flavoured food by a hollow square.

#### STFP training and tests

STFP was performed as illustrated in the schematic of Fig. 1a,b, with habituation, training and food-choice test sessions as described^7,14,39,59–64^, using two odour pairs (cinnamon versus cocoa^2,13,59^ or cumin versus thyme^38^). During habituation, both demonstrator (blue cartoon for cinnamon and yellow green for cumin) and observer mice (subject, brown cartoon) were singly housed in new cages with food deprivation for 12–15 h. Before STFP training, demonstrator mice were fed 1% cinnamon-flavoured or 0.5% cumin-flavoured food pellets for one hour. Only demonstrators that consumed more than 0.2 g food were used in subsequent STFP training sessions. Afterwards (during STFP training), demonstrator mice were allowed to socially interact with observer mice for 30 min in the absence of food. Food-choice tests (STFP memory tests) were performed immediately after STFP training and/or later as described in the figures with the observer mice that had been continuously single-housed and had been food-deprived for 12–15 h before the tests. In the food-choice tests, mice were offered cinnamon- and cocoa-flavoured or cumin- and thyme-flavoured food pellets for one hour. The food pellets were weighed before and after food-choice tests and the percentage of flavoured food eaten by observer mice was calculated. In all figures, data from three-week food-choice tests were shaded in grey to differentiate from the day 0 food-choice test data. The memory retention index was calculated as the ratio of the per cent cued food eaten in the 24-h, 48-h or 3-week food-choice test to the cued food eaten in the day 0 test. For the behaviour conducted in Extended Data Fig. 1b, observer mice were directly exposed to 1% cinnamon odour for 10 min. For Extended Data Fig. 1c, observer mice socially interacted with a toy demonstrator scented with 1% cinnamon food powder instead of a real demonstrator.

Note that in standard experiments (except where noted otherwise), observer mice were subjected to a food-choice test immediately after the training session (day 0 test). Observer mice were considered to be successfully STFP trained when the consumed cued food percentage exceeded 50%, and mice that did not learn the food odour as documented in the day 0 test were excluded from further analyses (except for the experiments in which memory acquisition was examined (Fig. 2a,e) or no day 0 test was performed (Extended Data Fig. 2c)). The success rate of STFP training was 70–90% for the cocoa and cinnamon odour pair, and around 50% for the cumin and thyme odour pair^38^ (Extended Data Fig. 2r). After the day 0 test, observer mice underwent only one additional food-choice test, at 24 h, 48 h or 3 weeks (‘STFP test’ in all schematics), with the following exceptions: for the experiments in Extended Data Fig. 1b,c,e, multiple food-choice tests were performed, whereas for the experiments in Extended Data Figs. 1d and 2c, no day 0 test was performed because these experiments aimed to ensure that the day 0 test did not influence long-term memory formation. In experiments using conspecific interaction controls or uncued food controls, demonstrator mice were fed with unscented food pellets but the procedure was otherwise the same.

#### Social behaviour during STFP training

Social behaviour during STFP training was recorded and analysed as described^2,36,65^. Observers’ sniffs of the demonstrator’s muzzle, body and anogenital region, as well as self-grooming bouts and fighting bouts, were annotated on a frame-by-frame basis using a MATLAB code BehaviorAnnotator (https://github.com/pdollar/toolbox). Pearson correlation analyses were performed between the behaviours scored and the percentage of consumed cued food^15,66^.

#### Buried food test

After food deprivation for 15–18 h, a test mouse was put into the centre of a new cage. A single normal food pellet was buried 1 cm under the bedding in a random corner. The latency the test mouse took to find the food pellet was video-recorded and measured offline^13^. The assay was finished within 5 min, so the latency for a test mouse that failed to dig up the pellet was recorded as 300 s.

#### Olfactory preference test

A 2 × 2-cm filter paper scented with water, 2-phenylethanol (10%, v/v) or 2-methylbutyric acid (10%, v/v)^67^ was sequentially provided to a test mouse after habituation. Each scented filter paper was placed in the cage at the opposite side of the test mouse for 3 min. The mouse behaviour was video-recorded and the total investigation time of the filter paper was scored blindly. The water-scented filter paper result was subtracted as the baseline from the total investigation time for the other two odours^68^.

#### Olfactory sensitivity test

A 2 × 2-cm filter paper containing a series of dilutions of isoamyl acetate in water (0, 0.001%, 0.01% and 0.1%) was placed in the opposite corner of the test mouse in a cage after cage habituation for 3 min. The sniffing time of each test mouse as a measurement of exploratory behaviour was video-recorded and quantified offline^68^.

#### Mapping odour-sensitive neurons using FOS staining

B6 mice were exposed to water, 2-phenylethanol or 2-methylbutyric acid applied to a 2 ×2-cm filter paper for 3 min, and then returned to their home cage. Ninety minutes after odour exposure, mice were anaesthetized and perfused. Brain slices from the mice were immunostained for FOS.

#### Non-associative olfactory memory

Non-associative olfactory memory was analysed as described^13^. In brief, cinnamon extract or anise extract was mixed with distilled water to a final concentration of 1%. On day 1, mice were allowed to freely sniff the odours in the chamber of the open field test used above for 10 min as an initial preference test. On day 2, mice were first exposed to the cinnamon odour in their home cage for 15 min. Then, after 30 min, the mice were returned to the chamber with the anise and cinnamon odours in two different random corners for another 10 min of sniffing. The anise preference index was calculated by dividing the investigation time of anise by that of cinnamon. The non-associative memory index was calculated by dividing the anise preference index of the second day (pre-exposure) by that of the first day (naive). Behaviour was recorded using the Viewer III tracking system, and analysed on a frame-by-frame basis using a MATLAB code BehaviorAnnotator (https://github.com/pdollar/toolbox) according to the previous description.

#### Fear conditioning

Fear conditioning experiments were conducted to evaluate contextual memory^33,54^. On day 1, the test mouse was trained in the fear conditioning chamber by pairing a 30-s, 80-dB, 2-kHz tone with a 2-s, 0.75-mA foot shock. On day 2, contextual memory was tested by placing the mice back into the fear conditioning chamber for 5 min. On day 3, altered context and tone tests were performed in a modified chamber in which the walls and the chamber bottom were covered with plastic sheets with colourful paintings or stripes. The test mouse was placed in the modified chamber for 5 min to measure altered context memory, followed by 1 min of tone (80 dB, 2 kHz) to measure the tone-associated memory. All behaviour was video-recorded and ‘freezing’ was quantified using FreezeView software (Coulbourn Instruments).

#### Open field tests

Open field tests were performed by placing a test mouse in the centre of a 40 × 40 × 40-cm open field box. The test mouse was given 15 min for free exploration. Behaviour was video-recorded and analysed using a BIOBSERVE III tracking system. The centre zone was defined as the central 20 × 20-cm square of the box centre manually during analysis, and the total distance travelled and time spent exploring the centre area were measured.

#### Three-chamber social behaviour

Three-chamber social behaviour was performed as described^33^. In brief, control and test mice expressing DREADDs or GFP were intraperitoneally injected with CNO 40 min before the test. Mice were placed at first in the central chamber to freely investigate all three chambers for 10 min. During the subsequent sociability test, a sex- and age-matched stranger mouse (stranger 1) was placed inside an upside-down wire pencil cup in one of the side chambers and an empty cup in the other side, and the exploratory behaviour of the test mouse was video-recorded for 10 min. During the following social novelty test, a second stranger mouse (stranger 2) was placed into the empty pencil cup of the three-chamber set-up and the exploratory behaviour of the test mouse was again video-recorded for another 10 min. The time mice spent in each chamber was analysed using the BIOBSERVE III tracking system.

### Slice electrophysiology

#### Slicing

Mice were anaesthetized using isoflurane, and brains were quickly removed into an ice-cold sucrose-based cutting solution (in mM: 228 sucrose, 26 NaHCO_3_, 11 glucose, 2.5 KCl, 1 NaH_2_PO_4_, 0.5 CaCl_2_ and 7 MgSO_4_, oxygenated by 95% O_2_ and 5% CO_2_). Coronal brain slices (300 μm) containing the COApm were sectioned with a vibratome (VT1200S, Leica Biosystems) and recovered in oxygenated artificial cerebrospinal fluid (ACSF, in mM: 119 NaCl, 26 NaHCO_3_, 2.5 KCl, 10 glucose, 1 NaH_2_PO_4_, 2.5 CaCl_2_ and 1.3 MgSO_4_) first at 32 °C for 30 min and then at room temperature for another 1 h. Slices were afterwards transferred to an electrophysiological recording chamber in which they were perfused with ACSF at 1 ml per min at 32 °C.

#### Optogenetic recordings

For verification of monosynaptic connections between AOB inputs and COApm neurons, 1 μM TTX and 100 μM 4-AP were included in the ACSF, and recordings were done as described^33^. The COApm was visualized with an upright microscope (Olympus, BX51WI) under a 60× water immersion objective. A 473-nm blue laser light was delivered to the COApm for 1 ms through a customized digital micromirror device-based photostimulation optogenetic system^33^. Layer 2 and layer 3 were distinguished from the distance to layer 1 and the intensity of neurons. Layer-2 tdT^+^, layer-2 tdT^−^, layer-3 tdT^+^ and layer-3 tdT^−^ neurons were all recorded through whole-cell voltage-clamp recordings. Glass pipettes (2–3 MΩ) were filled with internal solutions (in mM): 135 CsCl, 1 EGTA, 10 HEPES, 4 ATP-Mg, 0.1 spermine, 0.3 GTP-Na and 7 phosphocreatine (pH 7.2–7.30, osmolarity adjusted to 300–310). Picrotoxin (PTX; 50 μM), 50 μM APV and 20 μM CNQX were sequentially added in ACSF to determine whether light-evoked postsynaptic currents were inhibitory or excitatory. Neurons were clamped at −70 mV during recordings.

#### Optogenetic analyses of AMPAR-mediated synaptic plasticity

For optogenetic analyses of AMPAR-mediated synaptic plasticity, the following internal solution was used (in mM): 135 CsMeSO_3_, 1 EGTA, 10 HEPES, 4 ATP-Mg, 0.3 GTP-Na, 0.1 spermine and 7 phosphocreatine (pH 7.2–7.30, osmolarity adjusted to 300–310). In the AMPAR/NMDAR ratio experiment, 1 μM TTX, 100 μM 4-AP and 50 μM PTX were added in ACSF. Cells were held at −90 mV and given a 1-ms blue-light photostimulation to record AMPAR responses and then switched to +40 mV to record NMDAR responses. The peak of NMDAR-dependent light-evoked responses was measured at 50 ms after the onset of currents. The AMPAR/NMDAR ratio was calculated as NMDAR currents divided by the AMPAR currents. In AMPAR rectification experiments, 50 μM PTX and 50 μM APV were included in the ACSF with 1 μM TTX and 100 μM 4-AP. Blue-light-evoked AMPAR currents were recorded at −70 mV, 0 mV and +40 mV, respectively. The rectification index was calculated by absolute values of AMPAR currents at −70 mV divided by AMPAR currents at +40 mV.

#### Intrinsic excitability recordings

For intrinsic excitability recordings, whole-cell current-clamp recordings were achieved in layer-3 tdT^+^ neurons using the following internal solution (in mM): 135 K-gluconate, 10 HEPES, 0.25 EGTA, 1 MgCl_2_, 4 ATP-Mg, 0.3 GTP-Na, 0.1 spermine and 7 phosphocreatine (pH 7.2–7.30, osmolarity adjusted to 300–310). PTX (50 μM), 20 μM CNQX and 50 μM APV were included in the ACSF to block synaptic transmission^69^. After whole-cell recordings were established under voltage clamp, cells were switched to current clamp. Depolarizing currents from 0 pA to 250 pA (stepped by 50 pA, 1 s) were injected, and action potentials were recorded under current clamp. The current–frequency relationship was fitted with a single exponential equation^70^ in a transformed version: frequency = *a* × log_10_(current injections) − *a* × log_10_(*I*_0_), where *I*_0_ is the minimal current to elicit spikes. We calculated input resistances using Ohm’s law. Specifically, we injected currents ranging from −200 pA to +50 pA in 50-pA steps into neurons under current clamp and recorded the resulting voltage changes. The slope of the current–voltage relationship was then calculated as the input resistance. Resting membrane potentials were monitored after the stable establishment of whole-cell recordings without current injections. Action potential properties were analysed using parameters previously reported^30^.

#### Mini event recordings

To verify TeNT efficiency, mice were euthanized one week after virus injection and miniature EPSCs (mEPSCs) were monitored for 5 min in acute COApm brain slices in the presence of 1 μM TTX and 50 μM picrotoxin.

All junction potentials were not corrected. Cells were rejected for further analysis if series resistances changed more than 20% during recordings. All electrophysiological data were recorded using the MultiClamp 700B amplifier, digitalized at 10 kHz with Digidata1440, with Clampex 10.4, and analysed with Clampfit 10.4 (Molecular Devices).

### Biocytin labelling

During whole-cell recordings, biocytin (2 mg ml^−1^, Sigma, B4261) was included in CsCl-based internal solutions^71^. After recordings, recording electrodes were removed slowly and slices were immediately fixed in ice-cold 4% paraformaldehyde (PFA)/phosphate-buffered saline (PBS) solutions. Slices were washed in PBS for 5 min three times and then permeabilized and blocked in blocking buffer (containing 5% goat serum + 0.3% Triton X-100 in PBS) at room temperature for 1 h. Then Streptavidin Fluor 647 conjugate (S21374, Invitrogen, 1:1,000) was added for 2 h incubation at room temperature. Slices were then washed in PBS for 15 min, repeated four times, and were moved to PBS with DAPI (Sigma, D8417) to stain for another 15 min. After staining was done, slices were mounted onto Superfrost Plus slides with mounting medium (Fluoromount-G, 0100-01, SouthernBiotech). Images were taken with a Nikon confocal microscope (A1Rsi, Nikon, Japan) equipped with a 60× oil objective.

### Immunohistochemistry

Mice were deeply anaesthetized with isoflurane and transcardially perfused by PBS followed by ice-cold 4% PFA in PBS. For staining with anti-glutamate and anti-GABA antibodies, brains were placed into 30% sucrose/PBS solutions for cryoprotection without post-fixations. Otherwise, brains were post-fixed in 4% PFA overnight and switched into 30% sucrose/PBS solutions for another two days before further processing. Coronal COApm sections and sagittal AOB sections (both 40 μm thickness) were cut with a Lecia CM3050-S cryostat and incubated first at room temperature for 1 h in a blocking solution (5% goat serum and 0.3% Triton X-100 in PBS) and then at 4 °C overnight with primary antibodies (anti-glutamate, rabbit polyclonal, 1:1,000, Sigma-Aldrich G6642; anti-GABA, rabbit polyclonal, 1:1,000, Sigma-Aldrich A2052; anti-NeuN, mouse monoclonal, 1:1,000, Millipore, MAB377; anti-GFP, rabbit polyclonal, 1:1,000, Invitrogen A11122; anti-mCherry, rat monoclonal, 1:1,000, Invitrogen M11217; anti-FOS, Synaptic System 226308, guinea pig, 1:1,000). After 3× 15 min washing in PBS, sections were incubated with fluorescent secondary antibodies (goat anti-rabbit Alexa Fluor 488, Thermo Fisher Scientific, A11034; goat anti-rat Alexa Fluor 546, Thermo Fisher Scientific, A11081; goat anti-mouse Alexa Fluor 647, Thermo Fisher Scientific, A21236; for biocytin labelling, Streptavidin Fluor 647 conjugate, S21374, Invitrogen) in blocking buffer for 2 h at room temperature, washed 4× 15 min in PBS stained for 15 min with DAPI (Sigma, D8417) and mounted onto Superfrost Plus slides with mounting medium (Fluoromount-G, 0100-01, SouthernBiotech) for imaging.

### SynaptoTag tracing of efferent synaptic connections from the AOB

Three SynaptoTag constructs (non-floxed SynpatoTag, Cre-on SynaptoTag, and Cre-off SynaptoTag) were used. AAVs of these constructs were injected into the COApm either without or with prior injection of retro-AAVs encoding Cre recombinase into the AOB of six-to-eight-week-old wild-type C57BL/6J mice. Whole-brain coronal sections (40 μm) were collected from the beginning of the olfactory bulb to the end of the cerebellum six to eight weeks after injections. Every fifth section was stained with anti-GFP and anti-mCherry, mounted onto the Superfrost Plus slides in a rostral to caudal sequence and imaged using a Slide scanner (Olympus, VS200 or BX61VS) with a 10× objective. Mice were included in the analysis only when the virus injection accurately targeted the COApm.

### Retrograde trans-synaptic pseudotyped rabies virus tracing

Cell-specific monosynaptic rabies tracing was performed as described^50,72^. A 1:1 volume mixture of AAV5-CAG-DIO-TVA-mCherry (avian tumour virus receptor A) and AAV8-CAG-DIO-G (glycoprotein) was injected into the COApm (0.2 μl in total) unilaterally, whereas AAV2retro HA-tagged Cre was injected into the AOB of the same hemisphere of eight-week-old C57BL/6J mice. Two weeks after AAV injections, 0.2 μl of RVdG (GFP-tagged G-deleted rabies virus) was injected into the same COApm. Six days after RVdG injection, mice were perfused and fixed with PFA and their brains were analysed as described for the SynaptoTag mapping, using immunohistochemistry for GFP to detect input cells. All viruses used in rabies tracing were produced by the Janelia Farm Viral Core Facility.

### Imaging and image quantifications

Slides from the same experiments were imaged in parallel with the same settings using an Olympus Slide scanner. Quantifications of rabies and SynaptoTag tracings were performed as described^73^ with modifications. Brain regions were recognized under DAPI with the help of NeuroInfo Software (MBF Bioscience) under the guidance of the Franklin and Paxinos mouse brain atlas^74^ and the Allen Reference Atlas (https://atlas.brain-map.org/). For Fig. 1c and Extended Data Fig. 1i,j, the percentage of neurons was quantified by NeuN staining. For retrograde pseudotyped rabies virus tracings, cell bodies were counted manually with a cell counter. Input brain regions were presented as the percentage of GFP-positive cells among the total GFP-positive cells in the whole brain. For quantifications of presynaptic terminals using SynaptoTag (Syb2GFP), the averaged intensity of GFP signals of each brain region was measured by ImageJ and the background of each section was subtracted. To correct the variations caused by the different levels of virus injections and expression, the intensity of every brain region was normalized to the intensity of the injection site in each mouse’s COApm, which was identified by soma-expressed mCherry signals. For TRAP2 mapping, the cell layers of the COApm were delineated through the DAPI signal and the background of fluorescent channels. Cells labelled by tdTomato, GFP or both tdTomato and GFP, or DAPI only, were counted using the cell counter in ImageJ or CellProfiler. The percentage of activated cells among projection neurons was calculated as tdT^+^ and GFP^+^/total GFP^+^ cells × 100, and the percentage of activated cells in nonprojection neurons was calculated as tdT^+^ and GFP^−^/(DAPI-labelled cell nuclei – GFP^+^ cells) × 100.

### scRNA-seq and data analyses

#### Single-cell dissociation and flow cytometry (FACS)

AAV2retro-hSyn-tdTomato viruses were bilaterally injected into the AOB two weeks before the experiments. On the experiment day, mice were treated as follows: (1) mice in the ‘odour group’ were exposed to 1% cinnamon odour on a filter paper and then given the cocoa and cinnamon food choice; (2) mice in the STFP group were subjected to general STFP protocols (see above); that is, were enabled to socially interact with demonstrator mice who consumed 1% cinnamon-flavoured food and were then given the cocoa and cinnamon food choice; (3) mice in the home-cage group were not subjected to odour or STFP treatment, but otherwise were processed in parallel with the other two groups, and given normal food chow instead of the cocoa- and cinnamon-flavoured food pellets. All mice were single-housed and fasted. Thirty minutes after treatments, mice were euthanized and single neurons from the COApm were dissociated and sorted by fluorescence-activated cell sorting (FACS) as described^48^. In brief, mice were anaesthetized with isoflurane and decapitated quickly. Brains were removed into ice-cold choline chloride-based ACSF (in mM: 110 choline chloride, 24 NaHCO_3_, 20 glucose, 1.3 NaH_2_PO_4_, 2.5 KCl, 0.5 CaCl_2_, 7 MgCl_2_, 3 sodium-pyruvate,1.3 sodium-ascorbate, 2 thiourea and 13.2 trehalose, oxygenated by 95% O_2_ and 5% CO_2_). Coronal brain slices (300 μm) were cut using a vibratome (VT1200S, Leica Biosystems). Brain slices containing COApm were collected, and COApm was dissected under a fluorescence dissection microscope as accurately as possible according to the boundaries of the COApm, guided by retrogradely expressed tdTomato. Microdissected COApm tissues were incubated at 34 °C in papain enzyme mix containing DNase (LK003150, Worthington) with 800 nM kynurenic acid for 20 min. The tissue was gently triturated with a P1000 pipette, repeated every 15 min three times or until fully dissociated. After dissociation, cell suspensions were centrifuged at 350*g* for 10 min at room temperature. The supernatant was discarded and cell pellets were carefully resuspended in 1 ml oxygenated EBSS (with 10% ovomucoid inhibitor, 4.5% DNase and 800 nM kynurenic acid) and centrifuged, and the cell pellets were washed with 1 ml ACSF including 0.1% RNAse inhibitor. A 70-μm cell strainer (Thermo Fisher Scientific, 352350) was used to remove debris. Cells were stained with Hoechst (1:2,000; H3570, Life Technologies) for 10 min, washed and resuspended in ACSF. Cells were kept on ice or at 4 °C before they were sorted by FACS using a Sony SH800 sorter directly into 384-well plates with lysis buffer containing oligodT. Singlets were selected on the basis of Hoechst signals, and all Hoechst-positive singlet cells were collected^48^ (see Supplementary Fig. 1 for sorting strategy). Cells were sorted at a low rate, but each plate was done within 25 min. After FACS, plates were sealed, centrifuged and immediately snap-frozen and stored at −80 °C until further processing.

#### Library preparation and sequencing

The library was prepared according to the Smart-seq-2 protocol in a 384-well format^75^. In brief, cDNA was amplified by 23 PCR cycles. A PicoGreen quantitation assay in the 384-well format was used to assess cDNA concentrations, which were normalized to around 0.4 ng µl^−1^ per sample automatically performed by the TPPLabtech Mosquito HTS and Mantis (Formulatrix) robotic platforms. An in-house Tn5 was used to prepare, pool and clean libraries. Libraries were then sequenced on a Novaseq instrument (Illumina) using 2× 100-bp paired-end reads and 2× 12-bp index reads with a 200-cycle kit. Averaged sample reads per cell were 1.5 million.

#### Bioinformatics and data analysis

First, sequences obtained from Novaseq were de-multiplexed using bcl2fastq. Next, reads were aligned to the mouse mm10 genome (with tdTomato sequences added) augmented with ERCC (External RNA Controls Consortium) sequences using STAR (v.2.7.10a)^76^. We determined gene counts using FeatureCounts (v.2.0.0)^77^. We used standard algorithms and procedures for cell filtering, feature selection, dimensionality reduction and clustering. Genes were removed if they appeared in fewer than five cells. Cells with fewer than 500 genes or with fewer than 150,000 reads were also removed. In addition, cells with more than 5% reads as ERCC, and more than 5% mitochondrial transcripts, were also excluded from analysis. We log-normalized counts for each cell and scaled using ScaleData if necessary and appropriate^48^. This resulted in a dataset of 3,315 total cells, including 1,694 neurons.

Cells were visualized using UMAP. First, we aligned the raw data from all groups using the first ten canonical components of the ‘canonical correlation analysis’ function from the Seurat package (v.4.9.9)^78^. Principal component analysis was performed on projected genes into the principal component space. Single-cell principal component scores and gene loadings for the first 30 principal components were computed. Seurat’s FindClusters and Runumap functions were then used to calculate two-dimensional UMAP coordinates^78^.

We performed DEG analysis in three dimensions by applying the Mann–Whitney *U*-test to various cell populations. We used a *P* < 0.01 and log_2_-transformed fold change (log_2_FC) > 1 in both the STFP versus odour and the STFP versus home-cage comparisons. First, we identified DEGs between tdT^−^ and tdT^+^ cells within neuron cluster 1, separately in the three groups. Second, we analysed DEGs of neuron clusters between groups—namely, odour versus home cage, STFP versus home cage and STFP versus odour. Next, we identified exclusive DEGs by removing DEGs that are also present in tdT^−^ neurons as well as the odour and home-cage conditions. In detail, we identified DEGs in comparisons of AOB-projecting (tdT^+^) neurons in the STFP and the odour-only conditions. We then removed DEGs that are also differentially expressed in non-AOB-projecting neurons, allowing the identification of changes that were specific to AOB-projecting neurons that are selectively essential for long-term STFP memory. We also removed DEGs that were differentially expressed between the odour-only and the home-cage conditions to ensure that DEGs were not a consequence of an odour experience. These criteria produced a set of ‘STFP-specific DEGs’ (Supplementary Table 4 and Fig. 5f). All raw *P* values were adjusted using Benjamini–Hochberg correction^66^. All graphs and analyses were generated and performed in R (v.4.2.2).

### MERFISH spatially resolved transcriptomics

MERFISH experiments were performed as described^79^. The same behavioural design as was used in the scRNA-seq experiment was applied in the MERFISH experiment with three groups: (1) home-cage group; (2) odour group; and (3) STFP group.

#### MERFISH gene selection

To determine the optimal genes for MERFISH, we combined insights from the scRNA-seq data and the relevant literature. Our strategy centred on pinpointing marker genes for specific cell types using a comparative approach. (1) Identification process: we used the Mann–Whitney–Wilcoxon test to compare each gene’s expression between cells of a target population and all other cells. We then adjusted the resulting *P* values for multiple-hypothesis testing, yielding FDR-adjusted *P* values. (2) Selection criteria: the gene must be expressed in a minimum of 30% of cells in the target population. It should have an FDR-adjusted *P* value smaller than 0.001. Its expression in the target population should be at least four fold higher than the average in non-target cells. The proportion of cells expressing the gene in the target population should be at least twice as high as in any other cell group. Marker genes were ranked on the basis of their expression fold change compared with non-target cells. (3) We retained the top five marker genes from each cell type for further consideration. Beyond this data-driven approach, we also incorporated established genes linked to microglia, astrocytes and oligodendrocyte precursor cells (OPCs), as found in the literature. Furthermore, DEGs associated with remote memory were included, culminating in a comprehensive panel of 336 genes. Probes were designed using the Vizgen platform.

#### Tissue processing for MERFISH

Mice were anaesthetized and euthanized, and their brains were quickly dissected and frozen in OCT and stored at −80 °C until sectioning. Ten-micrometre-thick coronal sections containing the OFC or COApm and ventral hippocampus were collected using a Leica CM3050-S cryostat and directly mounted onto MERSCOPE slides for MERFISH analyses. Four coverslips of tissues were collected per mouse.

#### Sample preparation and MERFISH imaging

Slides were processed according to the MERSCOPE protocol (Vizgen). Slides were first washed three times in PBS, then permeabilized in 70% ethanol at 4 °C for 18 h. Slides containing tissue sections were then washed with sample preparation wash buffer (PN20300001) and incubated with formamide wash buffer (PN 20300002) for 30 min at 37 °C. Next, slides were incubated with the gene panel mix (RNA probes) at 37 °C for 48 h for hybridization and washed twice for 30 min in formamide wash buffer at 47 °C to remove excess coding and poly-A-anchor probes. The sections were then cleared by embedding in 4% polyacrylamide gel, followed by treatment with clearing premix (PN 20300003) at 37 °C for 36 h. Sections were then washed twice in sample preparation wash buffer, stained with DAPI/PolyT for an additional 15 min, washed with formamide wash buffer, again washed in sample preparation wash buffer and loaded into the MERSCOPE flow chamber. Images were captured at both 20× and 63× magnifications.

#### MERFISH data processing and analysis

MERFISH imaging data were processed using the MERlin pipeline^80^ with cell segmentation using CellPose^81^. Decoded molecules were registered and assigned to each cell as a MERFISH data matrix for further analysis. The MERFISH matrix for each section was concatenated, normalized, log-transformed with Scanpy^82^ and integrated using Harmony^83^. Leiden^84^ clustering was applied to generate cell clusters. DEGs identified in comparisons between groups or between tdTomato-positive and tdTomato-negative excitatory neurons were assessed using the Mann–Whitney–Wilcoxon test.

### Statistics and reproducibility

All experiments and data analyses were performed on anonymized samples or anonymized animals, except for the viral tracing experiments, in which the experimental condition can be identified on the basis of the pattern of virus expression. For quantitative imaging experiments, the number of replicates of ‘representative images’ is the same as the number of replicates specified for the corresponding quantifications. For non-quantitative imaging procedures, the number of replicates is the same as in the corresponding analysis experiments, or, as for all experiments, the experiments were repeated at least three times. All images and numbers were checked for inadvertent duplications using duplication detection software, although in several instances the same numbers resulted in different experiments and were retained. Statistics tests were performed by Prism v.10 or SPSS v.28, or by R software (for the scRNA-seq and MERFISH analyses). We first checked all data, except for the scRNA-seq and MERFISH data, for normality distribution using the Shapiro–Wilk or Kolmogorov–Smirnov tests. Then, we checked the equality of variances of all data using the Brown–Forsythe test. Then, parametric or nonparametric tests were applied accordingly with post-hoc tests for multiple comparisons. When datasets passed normality and equal variances tests, parametric tests such as two-tailed paired or unpaired Student’s *t*-tests, one- or two-way ANOVA tests or repeated-measures ANOVA tests with Tukey post-hoc tests were applied. If these were failed, nonparametric tests, such as two-tailed unpaired Mann–Whitney or Wilcoxon matched-pairs signed-rank tests or Kruskal–Wallis tests with post-hoc two-stage linear step-up tests were applied, with the adjusted *P* value used to determine significance in the post-hoc two-stage linear step-up test. For two-way ANOVA tests, if the data were not normally distributed, they were first transformed to ensure that they were in a Gaussian distribution. For the two-tailed Student’s *t*-tests, effect size and 95% confidence interval were calculated related to the standard deviation. In Supplementary Table 5, Cohen’s *d* = (mean of group B − mean of group A)/pooled standard deviation. scRNA-seq and MERFISH data were processed and analysed in R.

All numerical data are expressed as means ± s.e.m. *P < 0.05, **P < 0.01 and ***P < 0.001 denote significance when comparing between groups or animals. #*P* < 0.05, ##*P* < 0.01 and ###*P* < 0.001 denote significance for within-animal comparisons. All statistics are indicated in the figure legends. Further details for statistics are provided in Supplementary Tables 5 and 6, including effect sizes and confidence intervals. All primary data are deposited in publicly accessible repositories (https://purl.stanford.edu/gy983cn1444).

### Inclusion and ethics statement

As mandated by state and federal law, we were careful not to discriminate against anyone on the basis of race, ethnicity, gender, sexual orientation, age, ability, religion, socioeconomic status, nationality or any other protected or non-protected characteristic, and all individuals who contributed to the data collection are listed as co-authors regardless of race, ethnicity, gender, sexual orientation, age, ability, religion, socioeconomic status and nationality. Author contributions are listed in the ‘Author contributions’ section. Individuals who provided reagents, materials, suggestions and advice or any other types of contributions are acknowledged in the ‘Acknowledgments’ section.

This study was a collaboration between the laboratories of T.C.S. and S.R.Q. at Stanford University. The roles and responsibilities of the laboratories were discussed before the initiation of the experiments and were updated in step with discoveries emerging from the study. All required institutional approvals were obtained for this study from various committees at Stanford University, including full approval of the animal experiments from the Administrative Panel on Laboratory Animal Care at Stanford University. Requisite guidelines and regulations for research procedures, including for all animal experiments, were strictly followed.

As detailed in our laboratory’s publicly available manual, we are committed to an environment that values diversity, equity and inclusion on all perspectives, experiences and identities. We respect individuals from all backgrounds and are dedicated to promoting ethical conduct during our research. We do not tolerate any form of discrimination, harm, or disrespect in all aspects and uphold the dignity of all individuals. Moreover, we uphold all principles of intellectual honesty, plagiarism prevention and responsible citation practices that apply to research.

### Reporting summary

Further information on research design is available in the Nature Portfolio Reporting Summary linked to this article.

## Online content

Any methods, additional references, Nature Portfolio reporting summaries, source data, extended data, supplementary information, acknowledgements, peer review information; details of author contributions and competing interests; and statements of data and code availability are available at 10.1038/s41586-024-07632-5.

## Supplementary information

Supplementary Information Supplementary Discussion and Supplementary Figure 1

Reporting Summary

Supplementary Table 1 Supplementary Table 1 shows the Pearson correlation analyses between social behaviours and cued food consumed in Extended Data Fig. 2l.

Supplementary Table 2 Supplementary Table 2 shows the genes chosen for the MERFISH studies.

Supplementary Table 3 Supplementary Table 3 shows the STFP-specific gene values for volcano plot analyses in Fig. 4i and Extended Data Fig. 4l.

Supplementary Table 4 Supplementary Table 4 shows the value and percentage of STFP memory-specific genes in scRNA-seq analyses in Fig. 5f.

Supplementary Table 5 Supplementary Tables 5 and 6 are the statistics details for data analyses except for scRNA-seq and MERFISH.

Supplementary Table 6 Supplementary Tables 5 and 6 are the statistics details for data analyses except for scRNA-seq and MERFISH.

## Source data

Source Data Fig. 1

Source Data Fig. 2

Source Data Fig. 3

Source Data Fig. 6

Source Data Extended Data Fig. 1

Source Data Extended Data Fig. 2

Source Data Extended Data Fig. 3

Source Data Extended Data Fig. 9

Source Data Extended Data Fig. 10

## Data Availability

All primary data have been deposited in publicly available databanks, as follows. The scRNA-seq data are available through the Gene Expression Omnibus with accession numbers GSE256522 (for the new COApm data) and GSE152632 (for the previously published PFC data^48^). The MERFISH data are available through figshare at 10.6084/m9.figshare.25135124 (ref. ^85^). All other primary data are available at https://purl.stanford.edu/gy983cn1444. Source data are provided with this paper.

## Code availability

No custom code was generated for this study.
