# Supplementary material for: The cortical amygdala consolidates a socially transmitted long-term memory
Source: Nature. 2024 Jul 3;632(8024):366–74. doi: 10.1038/s41586-024-07632-5 (PMC11306109; doi:10.1038/s41586-024-07632-5)
Supplement: Supplementary file 1 — Supplementary Discussion and Supplementary Figure 1 [file 41586_2024_7632_MOESM1_ESM.pdf]

nature portfolio

https://doi.org/10.1038/s41586-024-07632-5

Supplementary information

The cortical amygdala consolidates a socially transmitted long-term memory

In the format provided by the authors and unedited

Supplementary Discussion

(1) We used male mice in the current study, but it should be noted that STFP behaviors are sexually dimorphic. A study of a modified STFP paradigm suggests that females are better at adopting socially transmitted food information from conspecific feces than males[86], while orexin-A-producing neurons in the lateral hypothalamic area are more activated in females than in males after STFP training[20]. The effects of 5-HT3A receptor knockout manipulations or of administration of the stress hormone corticosterone on STFP memory formation differ between males and females[21,87]. The neuropeptides vasopressin and oxytocin have been implicated in reproductive and social behaviors[88], although they are not considered traditional sexual hormones. Studies show that vasopressin metabolites or their analogs and oxytocin modulate STFP memory in a dose- and recency-dependent manner[89-91]. On the other hand, more direct evidence can be summarized from studies that female mice in the proestrus cycle express better STFP memory[22,92,93], possibly via activation of a G protein-coupled estrogen receptor[94]. The above literature suggests that STFP may be more affected by hormonal conditions in female mice than in male mice. Another reason for a potentially different mechanism of COApm function in female mice is that the COApm may be sexually dimorphic. The COApm expresses high levels of estrogen receptors[95,96] and male-specific pheromone receptors in male rats[97]. COApm is activated upon species-specific urine stimulation[98,99] and has been suggested to play an essential role in sexual behaviors[4,100], but not in predatory behaviors[101]. Therefore, considering the sexually dimorphic hormone and pheromone receptors expression patterns of the COApm, we hypothesize that the COApm may be sensitive to the estrous cycle in female mice. As a result, studying STFP in female mice would require a large expansion of experiments in which mice at different stages in the estrous cycle are being separately investigated. Given the focus of our study on the principal mechanisms of STFP memory and not on the hormonal regulation of STFP, we chose to study only male mice but feel that future experiments investigating female mice as a function of the estrous cycle would be very informative. Such studies could then use our current results as a starting point.

(2) Using analyses of scRNAseq data, we asked how the COApm cellular composition differs from that of the prefrontal cortex (PFC) for which deep SmartSeq2 scRNAseq data are readily available[48]. Integrated analysis of the transcriptome from the two regions revealed a total of nine neuronal populations (Extended Data Fig. 7j). Similar to the observations obtained with MERFISH analyses, the major cell types were conserved between the COApm and PFC (integrated clusters 1-3 and 7, Extended Data Fig. 7k, m, o), but their compositions differ remarkably in that some types of neurons seem to be specific for one or the other cortical region. For example, integrated clusters C5, C8, and C9 (corresponding to clusters 2, 4, and 6 in the COApm analysis, Extended Data Fig. 7l, n, p) were more abundant in COApm, and integrated clusters C4 and C6 were only observed in the PFC but not the COApm (Extended Data Fig. 7q, r). In contrast to neurons, integrated analyses of PFC and COApm glia cell transcriptomes revealed consistent glia cell types (Extended Data Fig. 7i).

(3) Genes upregulated after STFP training in projecting neurons included *Prkcg*, a neuron-specific PKC isoform associated with food reward spatial learning and memory[102,103]; *Flrt1*, a synaptic cell adhesion molecule which binds to latrophilin adhesion-GPCRs and plays a fundamental role in synapse formation[71]; and *Lifr*, a cytokine receptor that regulates cell proliferation, differation and survival[104].

(4) Our data suggest that a surrogate or food odor only can't induce successful STFP learning, indicating a social context is essential (Extended Data Fig. 1b, c) [18]. When the demonstrator and observer were

physically divided by a transparent Plexiglas barrier (without holes), or when the food odor cue is presented together with a dead demonstrator or on the posterior end of the live demonstrator, the STFP memory can not be successfully established[59,105]. Together, these suggest that social context with a live demonstrator's breath is essential during this process. On the other hand, long-term memory can be similarly achieved when odor or surrogate is paired with carbon-disulfide (CS_2), suggesting the communication is partly communicated by the demonstrator mouse to the observer by CS_2 during STFP[8,18,29,106,107]. Since the COApm forms extensive and sometimes reciprocal connections with the piriform cortex, which is also the main recipient brain region of the MOB, we propose the piriform cortex provides the major input for contextual olfactory cues to the COApm from a broad scope.

Supplementary Figure 1

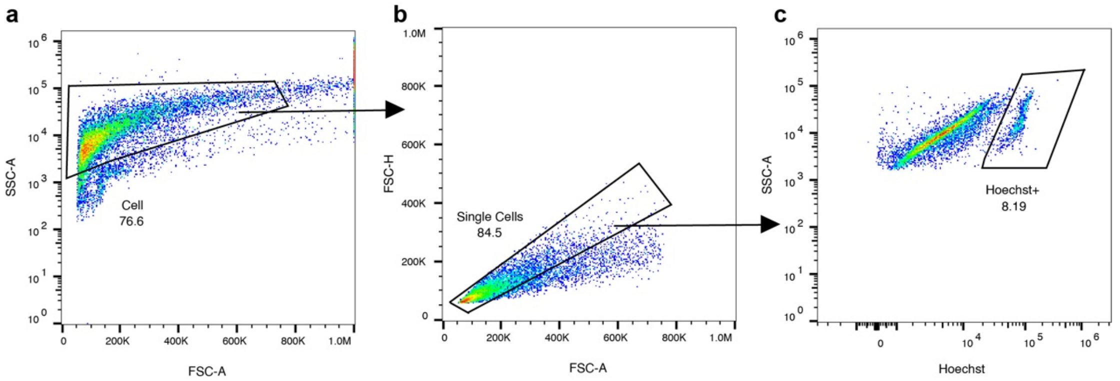

Supplementary Figure 1. Gating strategy for FACS (fluorescent-activated cell sorting) experiments. After identifying the cell population in the forward scatter area (FSC-A) vs. side scatter area (SSC-A) plot (**a**), doublets were eliminated by analyzing the cell population in the forward scatter area (FSC-A) vs. forward scatter height (FSC-H) plot (**b**). Then, Hoechst+ cells were selected for collection based on the Hoechst staining intensity (**c**).

Supplementary References:

86 Forestier, T., Feron, C., Raveleau, T., Sabatier, R. & Gouat, P. Transmission of food preference via faeces in male and female house mice: Who is a good provider of food cues? *Behav Processes* **179**, 104199 (2020).

87 Smit-Rigter, L. A., Wadman, W. J. & van Hooft, J. A. Impaired Social Behavior in 5-HT(3A) Receptor Knockout Mice. *Front Behav Neurosci* **4**, 169 (2010).

88 Donaldson, Z. R. & Young, L. J. Oxytocin, vasopressin, and the neurogenetics of sociality. *Science* **322**, 900-904 (2008).

89 Bunsey, M. & Strupp, B. J. A vasopressin metabolite produces qualitatively different effects on memory retrieval depending on the accessibility of the memory. *Behav Neural Biol* **53**, 346-355 (1990).

90 Strupp, B. J., Bunsey, M., Bertsche, B., Levitsky, D. A. & Kesler, M. Enhancement and impairment of memory retrieval by a vasopressin metabolite: an interaction with the accessibility of the memory. *Behavioral neuroscience* **104**, 268-276 (1990).

91 Popik, P. & Van Ree, J. M. Social transmission of flavored tea preferences: facilitation by a vasopressin analog and oxytocin. *Behav Neural Biol* **59**, 63-68 (1993).

92 Sanchez-Andrade, G., James, B. M. & Kendrick, K. M. Neural encoding of olfactory recognition memory. *J Reprod Dev* **51**, 547-558 (2005).

93 Clipperton, A. E., Spinato, J. M., Chernets, C., Pfaff, D. W. & Choleris, E. Differential effects of estrogen receptor alpha and beta specific agonists on social learning of food preferences in female mice. *Neuropsychopharmacology : official publication of the American College of Neuropsychopharmacology* **33**, 2362-2375 (2008).

94 Ervin, K. S., Mulvale, E., Gallagher, N., Roussel, V. & Choleris, E. Activation of the G protein-coupled estrogen receptor, but not estrogen receptor alpha or beta, rapidly enhances social learning. *Psychoneuroendocrinology* **58**, 51-66 (2015).

95 Simerly, R. B., Chang, C., Muramatsu, M. & Swanson, L. W. Distribution of androgen and estrogen receptor mRNA-containing cells in the rat brain: an in situ hybridization study. *The Journal of comparative neurology* **294**, 76-95 (1990).

96 Shughrue, P. J., Lane, M. V. & Merchenthaler, I. Comparative distribution of estrogen receptor-alpha and -beta mRNA in the rat central nervous system. *The Journal of comparative neurology* **388**, 507-525 (1997).

97 Guo, X., Guo, H., Zhao, L., Zhang, Y. H. & Zhang, J. X. Two predominant MUPs, OBP3 and MUP13, are male pheromones in rats. *Frontiers in zoology* **15**, 6 (2018).

98 Mucignat-Caretta, C. *et al.* Species-specific chemosignals evoke delayed excitation of the vomeronasal amygdala in freely-moving female rats. *Journal of neurochemistry* **99**, 881-891 (2006).

99 Pardo-Bellver, C., Martinez-Bellver, S., Martinez-Garcia, F., Lanuza, E. & Teruel-Marti, V. Synchronized Activity in The Main and Accessory Olfactory Bulbs and Vomeronasal Amygdala Elicited by Chemical Signals in Freely Behaving Mice. *Scientific reports* **7**, 9924 (2017).

100 Vinader-Caerols, C., Collado, P., Segovia, S. & Guillamon, A. Estradiol masculinizes the posteromedial cortical nucleus of the amygdala in the rat. *Brain research bulletin* **53**, 269-273 (2000).

101 Kondoh, K. *et al.* A specific area of olfactory cortex involved in stress hormone responses to

predator odours. *Nature* **532**, 103-106 (2016).
102 Douma, B. R., Van der Zee, E. A. & Luiten, P. G. Translocation of protein kinase Cgamma occurs during the early phase of acquisition of food rewarded spatial learning. *Behavioral neuroscience* **112**, 496-501 (1998).
103 Rossi, M. A., Mash, D. C. & deToledo-Morrell, L. Spatial memory in aged rats is related to PKCgamma-dependent G-protein coupling of the M1 receptor. *Neurobiology of aging* **26**, 53-68 (2005).
104 Nicola, N. A. & Babon, J. J. Leukemia inhibitory factor (LIF). *Cytokine Growth Factor Rev* **26**, 533-544 (2015).
105 Bennett G. Galef, J. & Stein, M. Demonstrator influence on observer diet preference: Analyses of critical social interactions and olfactory signals. *Animal learning & behavior* **13**, 31-38 (1985).
106 Munger, S. D. *et al.* An olfactory subsystem that detects carbon disulfide and mediates food-related social learning. *Current biology : CB* **20**, 1438-1444 (2010).
107 Maier, J. X., Blankenship, M. L., Barry, N. C., Richards, S. E. & Katz, D. B. Stability and flexibility of the message carried by semiochemical stimuli, as revealed by devaluation of carbon disulfide followed by social transmission of food preference. *Behavioral neuroscience* **128**, 413-418 (2014).
