# Supplementary material for: The cortical amygdala consolidates a socially transmitted long-term memory
Source: Nature. 2024 Jul 3;632(8024):366–74. doi: 10.1038/s41586-024-07632-5 (PMC11306109; doi:10.1038/s41586-024-07632-5)
Supplement: Supplementary file 2 — Reporting Summary [file 41586_2024_7632_MOESM2_ESM.pdf]

nature portfolio

Corresponding author(s): Zhihui Liu, Stephen R. Quake, Thomas C. Sudhof

Last updated by author(s): May 5, 2024

Reporting Summary

Nature Portfolio wishes to improve the reproducibility of the work that we publish. This form provides structure for consistency and transparency in reporting. For further information on Nature Portfolio policies, see our Editorial Policies and the Editorial Policy Checklist.

Statistics

For all statistical analyses, confirm that the following items are present in the figure legend, table legend, main text, or Methods section.

n/a	Confirmed	
☐	☒	The exact sample size (*n*) for each experimental group/condition, given as a discrete number and unit of measurement
☐	☒	A statement on whether measurements were taken from distinct samples or whether the same sample was measured repeatedly
☐	☒	The statistical test(s) used AND whether they are one- or two-sided<br>*Only common tests should be described solely by name; describe more complex techniques in the Methods section.*
☐	☒	A description of all covariates tested
☐	☒	A description of any assumptions or corrections, such as tests of normality and adjustment for multiple comparisons
☐	☒	A full description of the statistical parameters including central tendency (e.g. means) or other basic estimates (e.g. regression coefficient) AND variation (e.g. standard deviation) or associated estimates of uncertainty (e.g. confidence intervals)
☐	☒	For null hypothesis testing, the test statistic (e.g. *F*, *t*, *r*) with confidence intervals, effect sizes, degrees of freedom and *P* value noted<br>*Give P values as exact values whenever suitable.*
☒	☐	For Bayesian analysis, information on the choice of priors and Markov chain Monte Carlo settings
☒	☐	For hierarchical and complex designs, identification of the appropriate level for tests and full reporting of outcomes
☐	☒	Estimates of effect sizes (e.g. Cohen's *d*, Pearson's *r*), indicating how they were calculated

Our web collection on statistics for biologists contains articles on many of the points above.

Software and code

Policy information about availability of computer code

Data collection	-Clampex 10.4.0.36 data acquisition software (Molecular Devices)<br>-NIS-Elements AR 5.21.01 (Nikon)<br>-Olympus VS200 ASW 3.2.1 (Olympus)<br>-FreezeFrame (version 4)by Coulbourn Instruments<br>-Novaseq6000 (illumina)<br>-MERFISH images were collected with Vizgen MERSCOPE.
Data analysis	For behavior, imaging, and electrophysiological analysis:<br>-BIOBSERVE, Version 3.01 for behavior experiments.<br>-FreezeFrame (version 4)by Coulbourn Instruments for fear conditioning experiments<br>-Clampfit 10.4.0.36 for electrophysiological recordings<br>-NIS-Elements AR Analysis 5.21.01 (Nikon) for confocal image analysis<br>-Olympus OlyVIA 3.2.1, Fiji ImageJ (1.54b), and NeuroInfo 2021.1.5 for tissue imaging analysis<br>-GraphPad Prism 10 and SPSS 26 for statistical analysis<br>-CellProfiler (4.2.6)<br>For single-cell sequencing:<br>-R (version 4.2.2)<br>-Rstudio (version 2022.12.0+353)<br>-Seurat v4.9.9 by the Satija Lab (https://satijalab.org/seurat/).<br>For MERFISH imaging analysis:<br>-R(version 4.2.2) and Rstudio (version 2022.12.0+353)

-MERlin pipeline (2020 version, April version, git: Zhuanglab/MERlin), CellPose (2.0), Scanpy (l.9.1), Harmony (0.1.1) and Leiden (0.4.3) were applied.

For manuscripts utilizing custom algorithms or software that are central to the research but not yet described in published literature, software must be made available to editors and reviewers. We strongly encourage code deposition in a community repository (e.g. GitHub). See the Nature Portfolio guidelines for submitting code & software for further information.

Data

Policy information about availability of data

All manuscripts must include a data availability statement. This statement should provide the following information, where applicable:

- Accession codes, unique identifiers, or web links for publicly available datasets
- A description of any restrictions on data availability
- For clinical datasets or third party data, please ensure that the statement adheres to our policy

All primary data for this paper are deposited in publicly available databanks (single-cell RNAseq data accession numbers: GSE256522 [for the new COApm data reported here] and GSE152632 [for the previously published PFC data]; MERFISH data: https://doi.org/10.6084/m9.figshare.25135124; all other primary data are deposited in the Stanford Digital Repository (SDR; https://purl.stanford.edu/gy983cn1444).
Mouse mm10 genome was used in the scRNAseq analysis.

Research involving human participants, their data, or biological material

Policy information about studies with human participants or human data. See also policy information about sex, gender (identity/presentation), and sexual orientation and race, ethnicity and racism.

Reporting on sex and gender	N/A
Reporting on race, ethnicity, or other socially relevant groupings	N/A
Population characteristics	N/A
Recruitment	N/A
Ethics oversight	N/A

Note that full information on the approval of the study protocol must also be provided in the manuscript.

Field-specific reporting

Please select the one below that is the best fit for your research. If you are not sure, read the appropriate sections before making your selection.

☒ Life sciences ☐ Behavioural & social sciences ☐ Ecological, evolutionary & environmental sciences

For a reference copy of the document with all sections, see nature.com/documents/nr-reporting-summary-flat.pdf

Life sciences study design

All studies must disclose on these points even when the disclosure is negative.

Sample size	No statistical methods were used to predetermine sample sizes. Sample sizes in behavior tests and electrophysiological recordings were based on work in previous publications (PMID28683263, PMID32369733). For imaging experiments, at least three animals per genotype or condition were used based on a previous publication's results (PMID26232228). For single-cell RNA sequencing experiment, 3-5 mice per group were sequenced. For MERFISH images, 4 mice per group per section were imaged. Sample sizes for transcriptomics experiments were based on previous work (PMID38326616, PMID33177708).
Data exclusions	In behavior experiments, animals in which virus injections or canule implantations missed the target brain region were excluded. In tracing experiments, animals with virus injections that missed the target brain region were excluded. In single-cell RNA sequencing experiment, genes were removed if they appeared in fewer than 5 cells. Cells with fewer than 500 genes or with less than 150,000 reads were also removed. In addition, cells with more than 5% reads as ERCC and more than 5% mitochondrial reads were also excluded from analysis.
Replication	All experiments were performed with at least three independent experimental replicates, not just pseudo-replicates.
Randomization	Animals were randomized by cage prior to surgeries or behavioral training. For example, for pre-training injections male B6 mice in a cage with five animals were randomly assigned to the hM4Di or GFP groups in a counterbalanced manner. For imaging studies, littermates or same-aged mice were randomly assigned to each condition.
Blinding	All experimenters were blinded to the identity of the mice or the samples analyzed for all experiments except for the tracing experiments in which the viruses can be identified by the observed infection patterns.

Reporting for specific materials, systems and methods

We require information from authors about some types of materials, experimental systems and methods used in many studies. Here, indicate whether each material, system or method listed is relevant to your study. If you are not sure if a list item applies to your research, read the appropriate section before selecting a response.

Materials & experimental systems

n/a	Involved in the study
☐	☒ Antibodies
☐	☒ Eukaryotic cell lines
☒	☐ Palaeontology and archaeology
☐	☒ Animals and other organisms
☒	☐ Clinical data
☒	☐ Dual use research of concern
☒	☐ Plants

Methods

n/a	Involved in the study
☒	☐ ChIP-seq
☐	☒ Flow cytometry
☒	☐ MRI-based neuroimaging

Antibodies

Antibodies used

Primary antibodies:
anti-glutamate, Rabbit polyclonal, 1:1000, Sigma-Aldrich G6642; anti-GABA, Rabbit polyclonal, 1:1000, Sigma-Aldrich A2052; anti-NeuN, mouse monoclonal clone A60, 1:1000, Millpore, MAB377; anti-GFP, Rabbit polyclonal, 1:1000, Invitrogen A11122; anti-mCherry, Rat Monoclonal 16D7, 1:1000, Invitrogen M11217.anti-Fos, Synaptic System 226308, Guinea pig monoclonal Gp108B5, 1:1000

Secondary antibodies:
For immunocytochemistry, goat anti-rabbit Alexa Fluor 488 1:1000 (Thermo Fisher Scientific, A11034), goat anti-rat Alexa Fluor 546 1:1000 (Thermo Fisher Scientific, A11081), goat anti-mouse Alexa Fluor 647 1:1000 (A21236). For biocytin labeling, Streptavidin Fluor™ 647 conjugate (S21374, Invitrogen, 1:1000) was used.

Validation

Above antibodies were validated in previous publications:
anti-Glutamate and anti-GABA: Shang et al., 2018, Nat Commun., 9(1), 1232
anti-GFP and anti-NeuN: Wang et al., 2020, Neuron, 107(1):144-157.e4
anti-mcherry: Zhang et al., 2016, Nat Neurosci., Dec;19(12):1733-1742
Streptavidin Fluor™ 647 conjugate: Liu et al., 2022, eLife, Apr 14;11:e70664
anti-Fos: Choi et al., Nat Cummun, 2023 Mar 24;14(1):1631

Eukaryotic cell lines

Policy information about cell lines and Sex and Gender in Research

Cell line source(s)	HEK293T (CRL11268)
Authentication	HEK293T cells were directly purchased from ATCC
Mycoplasma contamination	Mycoplasma testing is performed by the ATCC prior to distribution
Commonly misidentified lines (See ICLAC register)	None of the commonly misidentified lines were used in this study

Animals and other research organisms

Policy information about studies involving animals; ARRIVE guidelines recommended for reporting animal research, and Sex and Gender in Research

Laboratory animals

All mice except for the TRAP2 mice were directly purchased from The Jackson Laboratory (C57BL/6J wild-type mice (Jax stock#: 000664); Ai75 (stock#: 025106), Ai14 (Jax stock#: 007914), Sun1-sfGFP (Jax stock#: 030952), vGAT-Cre (Jax stock#: 028862), PV-Cre (Jax stock#: 008069), vGluT2-Cre (Jax stock#: 028863), SST-Cre (Jax stock#: 013044), and CAMKII-Cre (Jax stock#: 005359)) and were maintained and bred in house. TRAP2 mice 53 containing a heterozygous Fos2A-iCreER allele were a generous gift from Dr. Liqun Luo (Stanford) and were crossed with Ai75 or C57BL/6J mice as indicated. All mouse lines were maintained on a C57BL/6J background and only male mice were used for experiments. Mice with Fos2A-iCreER and Ai75 alleles were only used in behavioral tests as heterozygotes. Mice obtained from Jackson laboratory were acclimated in the Stanford animal facility for at least two weeks before behavioral studies. Only adult mice (age 8 to 12 weeks) were used. Mice were fed ad libitum on the diet of mouse chew from ENVIGO (T2918.15) throughout the study. Mice were housed in groups with up to five mice per cage on 12-hour light-dark cycles (7 am to 7 pm, light) before behavior experiments took place. Test mice were single-housed during and after STFP behavioral experiments until food choice tests were performed All behavior experiments were performed during the same circadian period. Animals are kept with ambient temperature at 70 +/- 2 F and humidity at 55% +/- 5%.

Wild animals	No wild animals were used in this study
Reporting on sex	Only male mice were tested in this study. Because female mice exhibit estrous-dependent changes in STFP behavior (see Supplementary Discussion (1)), their use would mandate a large increase in animal numbers for experiments.
Field-collected samples	No samples were collected in the field.
Ethics oversight	All animal experiments were performed according to protocols and husbandry conditions that were reviewed and approved by the Administrative Panel on Laboratory Animal Care at Stanford University under the guidelines of the National Institutes of Health for the care and use of laboratory animals.

Note that full information on the approval of the study protocol must also be provided in the manuscript.

Plants

Seed stocks	No plants were involved in this study
Novel plant genotypes	No plants were involved in this study
Authentication	No plants were involved in this study

Flow Cytometry

Plots

Confirm that:

☒ The axis labels state the marker and fluorochrome used (e.g. CD4-FITC).

☒ The axis scales are clearly visible. Include numbers along axes only for bottom left plot of group (a 'group' is an analysis of identical markers).

☒ All plots are contour plots with outliers or pseudocolor plots.

☒ A numerical value for number of cells or percentage (with statistics) is provided.

Methodology

Sample preparation	The COApm was dissected from vibratome brain slices (300 µm thickness). Single cells were obtained after papain-mediated dissociation (LK003150, Worthington) according to the kit's instructions. Briefly, microdissected COApm pieces were incubated at 34°C in the papain enzyme mixture (containing DNase) with 800 nM kynurenic acid for 20 minutes, then the tissues were gently repeatedly triturated with a P1000 pipette three times every 15 minutes until the cells were completely dissociated (generally approximately 12 triturations in total). After dissociation, cell suspensions were centrifuged at 350g for 10 minutes at room temperature. The supernatant was discarded and cell pellets were carefully resuspended in 1 ml oxygenated EBSS (containing 10% v/v ovomucoid inhibitor, 4.5% v/v DNase, both provided in the kit, and 800 nM kynurenic acid), centrifuged, and cell pellets were washed with 1 ml ACSF containing 0.1% RNAse inhibitor. A 70-µm cell strainer (Fisher Scientific, 352350) was used to remove debris. Cells were stained with Hoechst (1:2,000; H3570, Life Technologies) for 10 minutes, washed, and resuspended in ACSF. Cells were kept on ice or at 4°C prior to flow cytometry.
Instrument	Sony SH800
Software	Software provided with Sony SH800 by the company was used
Cell population abundance	Singlets of Hoechst+ cells were around 2% of total events
Gating strategy	Singlets were selected based on Hoechst signals, and all Hoechst positive singlet cells were collected. No gating was set up for tdTomato signals. In this study, flow cytometry was used as a tool to sort single cells into 384 plates for further Smart-seq2 sequencing based the Hoechst signals. We did not use it to sort specific cell populations.

☒ Tick this box to confirm that a figure exemplifying the gating strategy is provided in the Supplementary Information.
